# Enhanced and directional light emission from two-dimensional excitons using Mie voids

**DOI:** 10.1038/s41467-026-76273-1

**Published:** 2026-08-05

**Authors:** Avishek Sarbajna, Ganesh Ghimire, Ilia D. Breev, Xavier Zambrana-Puyalto, Cheng Xiang, Alexander Huck, Timothy J. Booth, Søren Raza

**Affiliations:** Department of Physics, Technical University of Denmark, Kongens Lyngby, Denmark

**Keywords:** Nanocavities, Nanophotonics and plasmonics, Materials for optics, Nanoscale materials

## Abstract

Controlling light emission at the nanoscale has important applications in solid-state lighting, displays, and quantum light sources. Achieving this control requires both enhanced local electromagnetic fields to boost emission intensity and engineered radiation patterns to direct photons efficiently. Mie voids, consisting of an air cavity surrounded by a high-index semiconductor, are particularly suited for this purpose because they expose their strongest fields in an accessible region for nearby emitters while supporting resonances that shape directional emission through interference. Here, we demonstrate an all-van der Waals nanophotonic platform that couples excitons in atomically thin WS_2_ to Mie void resonators formed in WSe_2_. Guided by electromagnetic simulations, we identify void geometries that maximize photoluminescence through synergistic enhancement of excitation and emission processes. We also introduce a two-step fabrication approach that leverages van der Waals assembly to independently control the void diameter and depth. Experimentally, we observe up to a 600-fold increase in photoluminescence intensity from monolayer WS_2_ placed on individual voids compared to unstructured WSe_2_, along with pronounced out-of-plane beaming of light that yields a forward-to-off-axis enhancement of 2.6 dB. These results establish Mie voids in van der Waals semiconductors as a versatile platform for controlling light-matter interactions at the nanoscale.

## Abstract (web summary)

Here, the authors leverage the accessible electromagnetic fields and resonant interference of Mie void resonators to enhance and direct light emission from excitons in two-dimensional semiconductors, establishing Mie voids as a platform for controlling light-matter interaction.

## Introduction

Controlling the emission of light at the nanoscale is central to modern photonics, with applications ranging from solid-state lighting to single-photon quantum sources. The ability to enhance the emission intensity and control the direction of emission opens routes to highly efficient and compact light sources. Optical nanoantennas provide a versatile means to achieve this control, offering subwavelength confinement of electromagnetic fields and precise manipulation of emission characteristics^1,2^. This control relies on two underlying mechanisms, namely the amplification of the local electromagnetic field experienced by the emitter and the tailoring of the far-field radiation pattern so that photons are efficiently directed toward the desired collection direction.

Early realizations of optical nanoantennas relied on metallic structures that confine light below the diffraction limit through plasmonic resonances^3–8^. While highly effective at boosting local fields, plasmonic antennas suffer from intrinsic absorption losses and often require complex geometries. To overcome these limitations, dielectric nanoantennas based on high-refractive-index materials have emerged as an attractive low-loss alternative^9–12^. These resonators support both electric and magnetic Mie resonances that can enhance photoluminescence (PL)^13–17^ and interfere with the emitter’s dipole radiation to enable directional emission^18–25^. However, in conventional dielectric Mie resonators, the strongest electromagnetic fields are confined inside the high-index material, which makes it difficult to overlap the field maximum with nearby emitters^26^. This limitation becomes even more pronounced because achieving optimal directionality typically requires either a significant emitter-resonator separation^27^ or the use of moderate-index materials^28,29^, both of which reduce the local field enhancement experienced by the emitter. Achieving simultaneous local-field amplification and directional emission, therefore, remains a central challenge.

A promising route to address this challenge is to invert the conventional Mie resonator geometry and form structures known as Mie voids^30,31^. These consist of a low-index region, typically air, surrounded by a high-index medium. Such complementary architectures support the same family of Mie resonances, namely electric and magnetic multipoles, but they expose their strongest electromagnetic fields inside the low-index region^32^. This geometry naturally places the electromagnetic field maximum in an accessible volume where an emitter can be positioned while maintaining the ability to tailor far-field emission through interference between the emitter and the void resonances. Since the optical confinement occurs within the void rather than the high-index host, Mie voids also allow the use of materials with high refractive index and moderate absorption, which further increases the index contrast and broadens the range of suitable materials^33–36^. These combined properties make Mie voids an appealing platform for achieving simultaneous local-field enhancement and directional light emission.

**Fig. 1:**
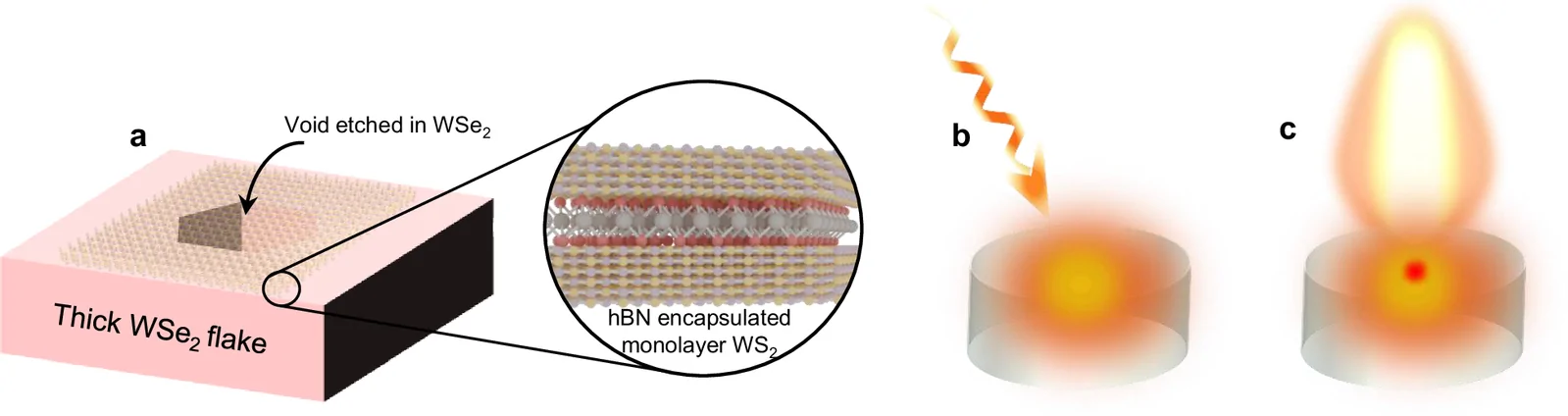
Controlling light emission using void resonators. **a** Schematic of the device displaying an hBN encapsulated monolayer of WS_2_ (zoomed) on top of a void resonator constructed in WSe_2_. **b** Field localization within the air region enabled by the void resonance. **c** Emitter-void interaction that results in enhanced and out-of-plane directional emission.

In this work, we demonstrate the potential of Mie voids for controlling light emission using an all-van der Waals nanophotonic platform (Fig. 1a). Electromagnetic simulations show that carefully tuning the void dimensions leads to pronounced enhancement of PL through two synergistic effects, namely strong field confinement inside the void (Fig. 1b) and enhanced out-of-plane emission enabled by emitter-void coupling (Fig. 1c). We verify these predictions experimentally and observe up to a 600-fold enhancement in the PL intensity of the A-exciton in WS_2_ coupled to individual voids at room temperature, compared to emission from unpatterned WSe_2_. To demonstrate directional emission, we measure the angular distribution of emitted light using back-focal-plane imaging of void arrays and find that the emission profile evolves from the Lambertian pattern of uncoupled emitters to a highly directional distribution with enhanced emission normal to the surface. The combination of field localization in air, directional emission, and emitter-mode alignment establishes a versatile framework for manipulating two-dimensional excitons using Mie voids.

## Results

### Designing Mie voids for maximal light emission

**Fig. 2:**
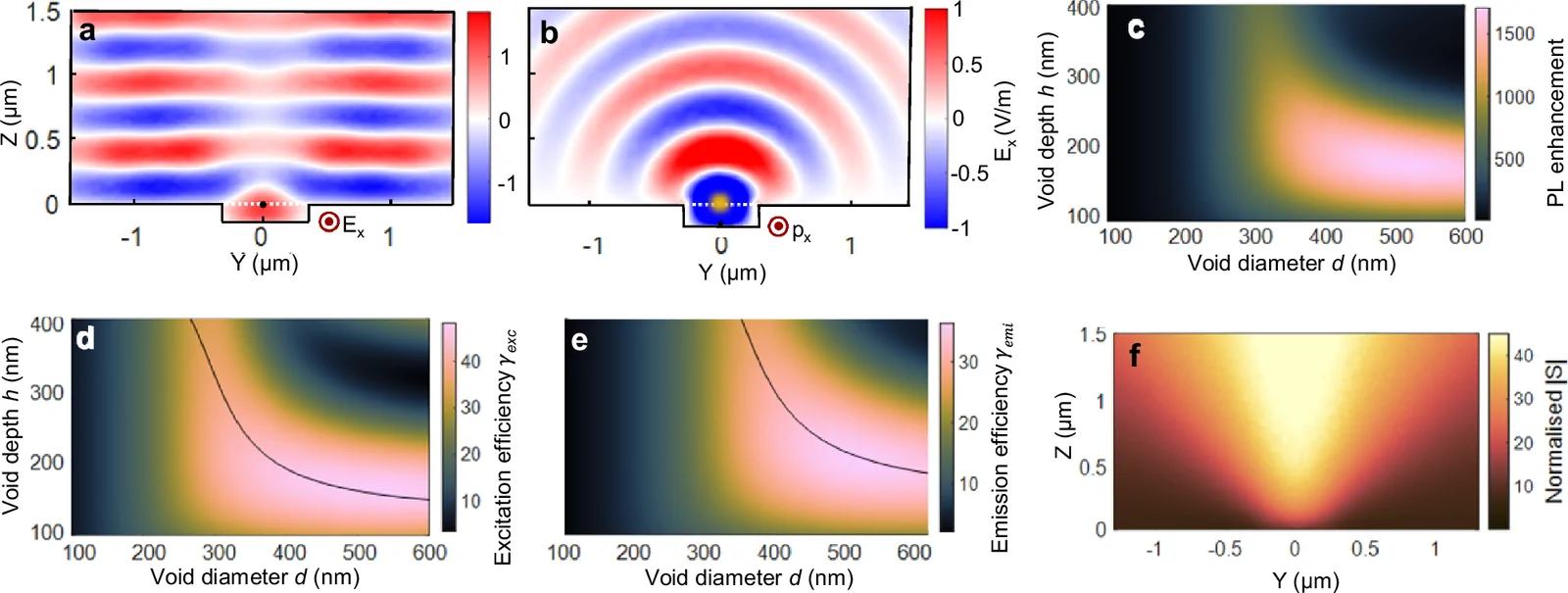
Simulation of enhanced and directional light emission. **a** Simulated electric field distribution *E*_*x*_ in the *yz*-plane for a void with diameter *d* = 540 nm and depth *h* = 160 nm. The excitation is a normally-incident plane wave polarized along *x* with a wavelength of *λ*_exc_ = 532 nm. The black border separates the air and WSe_2_ domains. The white dashed line marks the top surface of the void. The black dot denotes the center of the void opening, where the electric field intensity is evaluated to obtain the excitation efficiency. **b** Simulated electric field distribution *E*_*x*_ from an *x*-oriented dipole (yellow dot) emitting at a wavelength of *λ*_emi_ = 610 nm and placed at the center of the void opening. The void dimensions are *d* = 500 nm and *h* = 200 nm. **c** Photoluminescence enhancement as a function of void diameter *d* and depth *h* calculated using Eq. (1). **d**, **e** Excitation and emission efficiencies as a function of void dimensions, respectively. The black lines depict the depth vs. diameter relation given by Eq. (2). **f** Simulated Poynting vector amplitude of dipole-on-void system normalized by dipole on flat WSe_2_. The void dimensions are the same as in (**b**).

The PL signal from monolayer WS_2_ originates from an excitation process at a wavelength of *λ*_exc_ = 532 nm, where incident photons generate electron-hole pairs in the conduction and valence bands. Through primarily nonradiative relaxation, these carriers form A-excitons that recombine radiatively and emit light at *λ*_emi_ = 610 nm^37^. Both the excitation and emission processes can be enhanced by coupling the emitter to a Mie void resonator. To determine the void depth *h* and void diameter *d* that maximize the PL signal, we perform three-dimensional electromagnetic simulations using the finite-element method in COMSOL Multiphysics (see “Methods”). The excitation process is modeled by a plane wave normally incident on a single void (Fig. 2a), while the emission process is modeled by an electric dipole (ED) oriented parallel to the sample surface and positioned laterally at the center of the void and vertically at its opening (Fig. 2b), matching the experimental configuration in which the encapsulated WS_2_ monolayer rests on top of the WSe_2_ voids. We choose WSe_2_ as the host material because its high refractive index and non-negligible extinction coefficient in the visible lead to strong reflection at the void boundaries, enabling the formation of Mie void resonances^31^.

Enhancement of the excitation process *γ*_exc_ is quantified through the intensity of the electric field component along the dipole oscillation direction **p**_dp_ at the dipole position, i.e., ∣**E**(**r**_dp_, *λ*_exc_) ⋅ **p**_dp_∣^2^. Enhancement of the emission process *γ*_emi_ is characterized by the total radiated power integrated over the solid angle corresponding to the numerical aperture (NA) of the objective lens used experimentally. The emitted power is calculated from the Poynting vector **S** of the dipole field. The total PL enhancement is obtained by normalizing the simulated and measured PL intensity from the void resonator to that from a flat unstructured WSe_2_ region

1 $$P{L}_{{{\rm{enh}}}}=	 {\gamma }_{{{\rm{exc}}}}({\lambda }_{{{\rm{exc}}}})\times {\gamma }_{{{\rm{emi}}}}({\lambda }_{{{\rm{emi}}}})\\=	 \frac{{\left\vert {{{\bf{E}}}}_{{{\rm{void}}}}\left({{{\bf{r}}}}_{{{\rm{dp}}}},{\lambda }_{{{\rm{exc}}}}\right)\cdot {{{\bf{p}}}}_{{{\rm{dp}}}}\right\vert }^{2}}{{\left\vert {{{\bf{E}}}}_{{{\rm{flat}}}}\left({{{\bf{r}}}}_{{{\rm{dp}}}},{\lambda }_{{{\rm{exc}}}}\right)\cdot {{{\bf{p}}}}_{{{\rm{dp}}}}\right\vert }^{2}}\\ 	 \times \frac{\int_{{{\rm{NA}}}}{{{\bf{S}}}}_{{{\rm{void}}}}({\lambda }_{{{\rm{emi}}}})\cdot \hat{{{\bf{r}}}}\,d\Omega }{\int_{{{\rm{NA}}}}{{{\bf{S}}}}_{{{\rm{flat}}}}({\lambda }_{{{\rm{emi}}}})\cdot \hat{{{\bf{r}}}}\,d\Omega },$$

where $\hat{{{\bf{r}}}}$ denotes the unit vector in the radial direction.

Our simulations show that the PL enhancement strongly depends on the void dimensions. Figure 2c shows that a void with a depth of 180 nm and a diameter of 520 nm yields the highest enhancement, reaching a 1700-fold increase in PL compared to unpatterned flat WSe_2_ and around 60-fold increase compared to flat SiO_2_ substrate (Supplementary Section 1, Fig. S1). The larger enhancement relative to WSe_2_ arises because the high-index and absorptive substrate suppresses both the excitation and emission processes, leading to a quenched reference signal. We use flat WSe_2_ as the reference, as it provides a consistent experimental baseline within the same sample and reflects the high-index material platform required for realizing void resonators. Increasing the void depth moves the field maximum deeper into the cavity, thereby reducing the spatial overlap with the dipole at the surface and thus weakening their interaction (Supplementary Section 2, Fig. S2).

To disentangle the excitation and emission contributions, we separately compute their efficiencies as functions of the void dimensions (Fig. 2d, e). Both exhibit similar trends with changing void size, but their maxima occur under slightly different conditions. The excitation efficiency peaks for a void with a depth of *h* = 160 nm and diameter of *d* = 540 nm, while the emission efficiency reaches its maximum for a void with dimensions *h* = 200 nm and *d* = 500 nm. The difference in optimal dimensions arises because the excitation and emission enhancements occur at different wavelengths.

The depth-diameter trends in the excitation and emission efficiencies can be understood from a constructive-interference model (see Supplementary Section 3, Fig. S3). In this model, light entering the void is approximated as a plane wave that propagates with an effective wavenumber ${k}_{z}={k}_{0}{{\rm{Re}}}\left[{n}_{{{\rm{eff}}}}(d)\right]$, where *n*_eff_ is the effective index of the fundamental electric-dipolar mode of an air-WSe_2_ cylindrical waveguide and depends on the diameter (see Fig. S3). Maximal field enhancement at the void opening occurs when the downward-propagating wave, after reflection at the void bottom, returns in phase with the field at the aperture, which is satisfied when

2 $$2{k}_{0}{{\rm{Re}}}\left[{n}_{{{\rm{eff}}}}(d)\right]h+\phi=2\pi .$$

In Eq. (2), *k*_0_ = 2*π*/*λ* is the free-space wavenumber and *ϕ* is the phase accumulated upon reflection at the void bottom, which is close to *π* and nearly constant across all diameters (see Fig. S3). It should be noted that the same condition also governs the directionality of the emission, since constructive interference between the upward-emitted and downward-reflected waves enhances radiation into the top (+*z*) direction. This model quantitatively captures the depth-diameter relation observed in the full-wave simulations of the excitation and emission efficiencies, as shown by the overlaid lines in Fig. 2d, e. The overall PL enhancement is therefore maximized when both efficiencies coincide, underscoring that the enhancement arises from a synergistic interplay between optimized field localization and directional far-field emission. For comparison, analogous simulations for Si nanodisks show that the excitation and emission efficiencies often peak at markedly different geometries, in many cases remaining far apart in parameter space, resulting in lower and geometry-sensitive PL enhancement (Supplementary Section 4, Fig. S4).

To quantify directionality, we compute the Poynting vector amplitude for a dipole placed on a void resonator and normalize it to that of a dipole on flat WSe_2_ (Fig. 2f). The void-coupled configuration yields up to a 48-fold enhancement of the power flow, confirming that Mie voids efficiently direct emission into the out-of-plane direction.

### Fabrication with independent control of diameter and depth

**Fig. 3:**
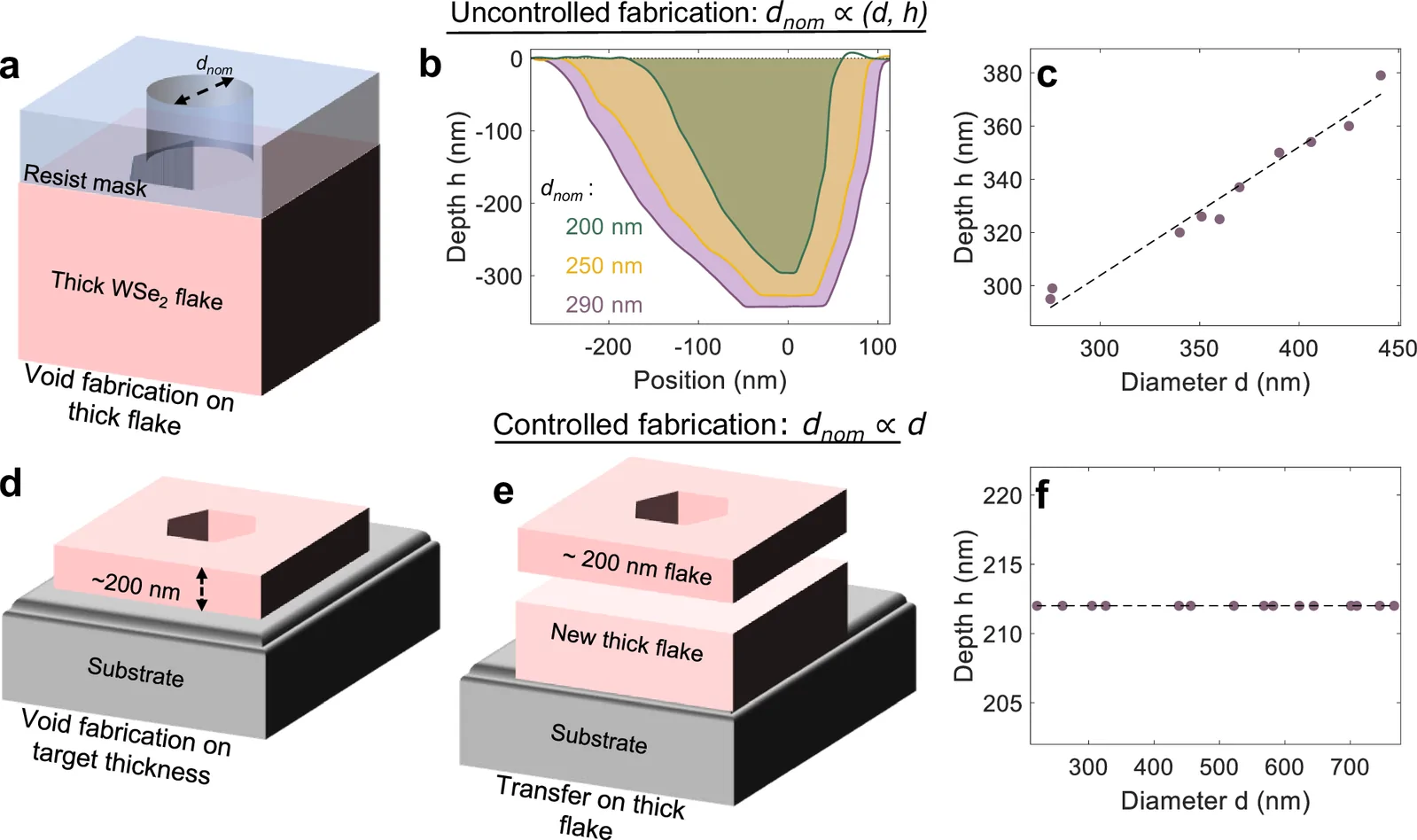
Solving fabrication barrier. **a** Schematic of conventional lithographic fabrication of Mie voids in a WSe_2_ flake (pink) with a thickness exceeding the targeted void depth. The etched void diameter can exceed the nominal diameter *d*_nom_ defined by the resist mask opening (blue) due to lateral etching. **b** Atomic-force microscopy measurements of voids fabricated using identical etch times show that larger mask openings *d*_nom_ produce voids with both larger diameters *d* and larger depths *h*. The surface is at *h* = 0 nm. **c** Measured void depth versus diameter for the conventional process, demonstrating a near-linear dependence on the void dimensions due to the mask-opening-dependent etch rate. **d**, **e** Mie voids fabricated using a two-step process to independently control the diameter and depth. A WSe_2_ flake with a thickness matching the target depth (~200 nm) is lithographically patterned (**d**). The patterned flake is subsequently transferred onto an optically-thick WSe_2_ that serves as a substrate to provide the bottom interface of the Mie void (**e**). **f** Measured void depth versus diameter for the two-step fabrication process. The void diameter is controlled by the mask opening, while the depth remains constant at *h* = 212 nm.

The electromagnetic simulations show that achieving maximal PL enhancement depends sensitively on the void dimensions (Fig. 2c). Consequently, it is crucial to independently control the void depth and void diameter in the fabrication process^38^. However, when performing lithographic patterning at the nanoscale, the etch rate can depend on the nominal diameter *d*_nom_, which is dictated by the size of the opening in the resist mask (Fig. 3a). In particular, lateral and vertical etch rates differ, with smaller nominal diameters typically exhibiting slower vertical etching. In addition, for arrays of voids, the concentration of voids can further accelerate or retard etching, complicating precise control over void dimensions (Supplementary Section 5, Fig. S5)^31,39–43^. This means that both the void diameter and void depth depend on the nominal diameter *d*_nom_ (Fig. 3b). Larger mask openings yield deeper and wider voids. Figure 3c quantifies the correlation between void depth and void diameter for samples etched for the same duration but with different nominal diameters. Similar observations have been reported in other materials and fabrication processes related to fabricating Mie voids, such as dry etching in GaAs^44^ and focused ion beam milling in Si^30^.

To decouple diameter and depth during fabrication, we develop a two-step process which leverages the layered nature of WSe_2_ to independently control the void diameter via the mask size and the depth via mechanical exfoliation and transfer techniques (see “Methods”). The first step is to exfoliate a WSe_2_ flake with a thickness that matches the target void depth (~200 nm). As the void depth *h* is dictated by this flake thickness, the opening in the resist mask now only controls the void diameter *d* (Fig. 3d). After lithographic patterning and etching, the second step is to transfer the thin flake with void resonators onto an optically-thick WSe_2_ substrate to provide the bottom interface of the void resonator (Fig. 3e). This two-step process decouples the two geometric parameters of the void resonators, enabling controlled fabrication of voids with different diameters at a constant depth (Fig. 3f). The depth of all the voids is *h* = 212 nm and the WSe_2_ substrate beneath the resonators has a thickness of 2.7 μm (see “Methods”). In the final step, we transfer an hBN-encapsulated monolayer of WS_2_ onto the voids. The hBN layers have a thickness of ~5−7 nm. The encapsulation protects the monolayer from environmental degradation, suppresses interlayer charge transfer and improves surface flatness^45,46^. We have conducted additional simulations to investigate whether the slight vertical displacement from the void opening due to the hBN encapsulation impacts the emission efficiency (see Supplementary Section 6, Fig. S6). These simulations show that the emission efficiency remains nearly constant even if the dipole is displaced by up to 20 nm above the surface, indicating that the thin hBN encapsulation has only a minor impact on the emission efficiency.

### Photoluminescence enhancement from individual Mie voids

**Fig. 4:**
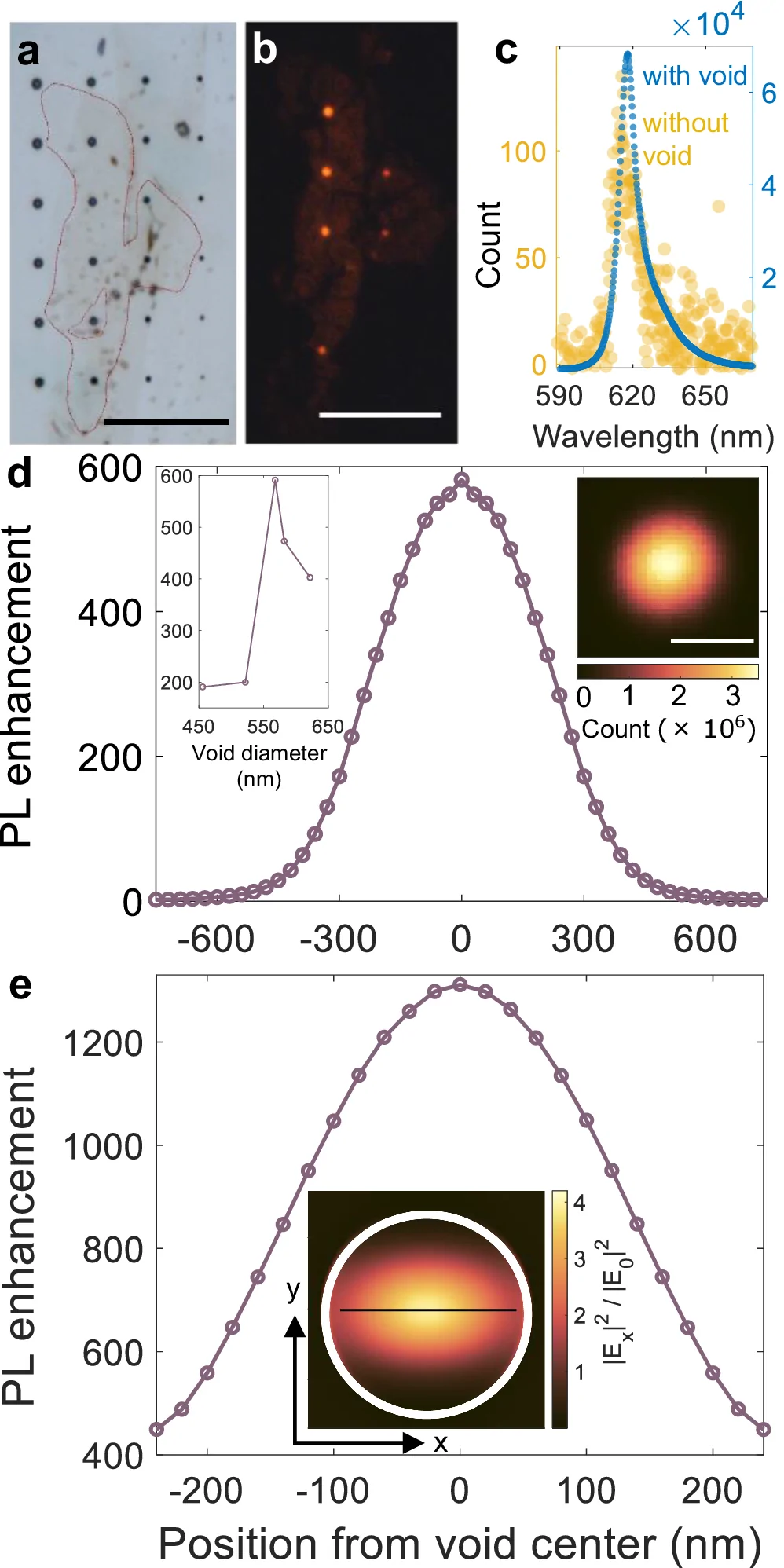
Void-enabled photoluminescence enhancement. **a** Bright-field micrograph of the WS_2_ monolayer on voids with different diameters. The red border indicates the monolayer. **b** Corresponding optical image of PL emission showing much brighter emission from the voids relative to flat WSe_2_. The scale bars for both the images are 10 μm. **c** Spectrally resolved PL signal with and without the void resonator. **d** Experimental PL enhancement of monolayer-on-void structure as a function of the position from the void center. Top-right inset shows two-dimensional PL mapping of the same structure (scale bar: 500 nm). Top-left inset shows experimental peak PL enhancement of voids with varying diameters. **e** Simulated PL enhancement as a function of the dipole position from the void center following the similar trend with the experimental result. The inset shows the normalized electric field profile at the void opening at an excitation wavelength of 532 nm. The black line indicates the axis along which the PL enhancement is calculated.

Figure 4a shows an optical micrograph of the encapsulated monolayer WS_2_ stacked on voids of different diameters. PL imaging reveals that the void regions appear significantly brighter than the adjacent flat WSe_2_ areas, indicating enhanced emission due to the void resonators (Fig. 4b). Spectrally-resolved PL measurements of the monolayer WS_2_ on both the void resonator and the flat WSe_2_ confirm that the observed emission arises from the A-exciton in monolayer WS_2_ (Fig. 4c). The PL peaks around a wavelength of 620 nm for the monolayer on the void and 615 nm for the monolayer sitting on flat WSe_2_, which are slightly red-shifted from the A-exciton wavelength of unencapsulated WS_2_ (~610 nm). The hBN encapsulation decreases the exciton binding energy due to dielectric screening, thereby causing a red-shift in the PL emission^47–50^.

We perform PL mapping using a confocal setup (see “Methods”) to spatially resolve the emission across the void (Fig. 4d). The excitation beam has a spot diameter of approximately 1 *μ*m. The sample is mounted on a piezo stage, and the map is acquired by recording the PL signal in translation steps of 30 nm across the targeted area. The upper-right inset of Fig. 4d shows a zoomed-in PL map of a WS_2_ monolayer stacked on top of a single void. The 1D intensity plot extracted from the map (Supplementary Section 7, Fig. S7) represents the PL enhancement. Benchmarking the void-assisted emission with that of emission from the flat WSe_2_ substrate, we observe up to 600-fold PL enhancement at the void center, for a void with dimensions *d* = 568 nm and *h* = 212 nm. We further see that the PL emission peaks at the void center and decays almost symmetrically toward the rim. Control experiments show that the observed enhancement predominantly arises from coupling to the void resonances rather than strain-induced effects (Supplementary Section 8, Fig. S8). We extend these measurements to voids of different diameters and track the peak PL enhancement (Fig. 4d, top-left inset). We observe that the enhancement increases with increasing diameter until it reaches an optimal size, beyond which further increase of the void diameter causes the enhancement to drop. When the void diameter exceeds the optimal size, the excitation and emission wavelengths detune from the void resonance wavelength, resulting in a weakening of the field localization and, consequently, the PL enhancement.

To understand the experimental results, we perform simulations of the spatially-resolved PL enhancement by translating a single dipole radially outward from the void center and calculate the emission efficiency at each position. By multiplying the spatially-resolved emission efficiencies with the normalized electric field intensities at the same positions extracted from the corresponding excitation efficiency simulations, we can evaluate the spatial dependence of the PL enhancement. Figure 4e shows the simulation result of the spatially-resolved PL enhancement for a void of identical dimensions to the experimental sample shown in Fig. 4d. The PL enhancement is maximal for a dipole positioned at the void center and decreases as the dipole shifts radially outwards, matching our experimental outcome. The simulated enhancement reaches around 1300×, more than 2 times higher than the experimental value. The lower experimental enhancement is due to the diffraction-limited spot size of the illumination laser, which excites an ensemble of excitons distributed across the entire void. This is different from the simulation model, which considers only a single dipole at each position. To account for this finite spot size, we convolved the simulated spatial PL profile with a Gaussian kernel corresponding to the experimental illumination spot, which reduces the effective peak enhancement at the void center to approximately 730× (see Supplementary Section 9, Fig. S9). Although the absolute peak values differ slightly, the spatial profile of the simulated PL enhancements is in good agreement with the experimental results. In addition, the spatial dependence of PL enhancement follows the electric field profile of the void resonance (Fig. 4e, inset). The apparent asymmetry between the *x*- and *y*-directions in the simulated field profile arises from the use of *x*-polarized excitation in the calculations. In contrast, the experimental illumination is unpolarized, which results in a radially symmetric response in the mapping. This confirms that the PL enhancement originates from the capability of the void resonance to enhance the local electric field and thereby enhance both excitation and emission efficiencies (see Supplementary Section 6 and Fig. S6 for spatially-resolved simulations of the excitation and emission efficiencies).

### Directional emission using Mie voids

**Fig. 5:**
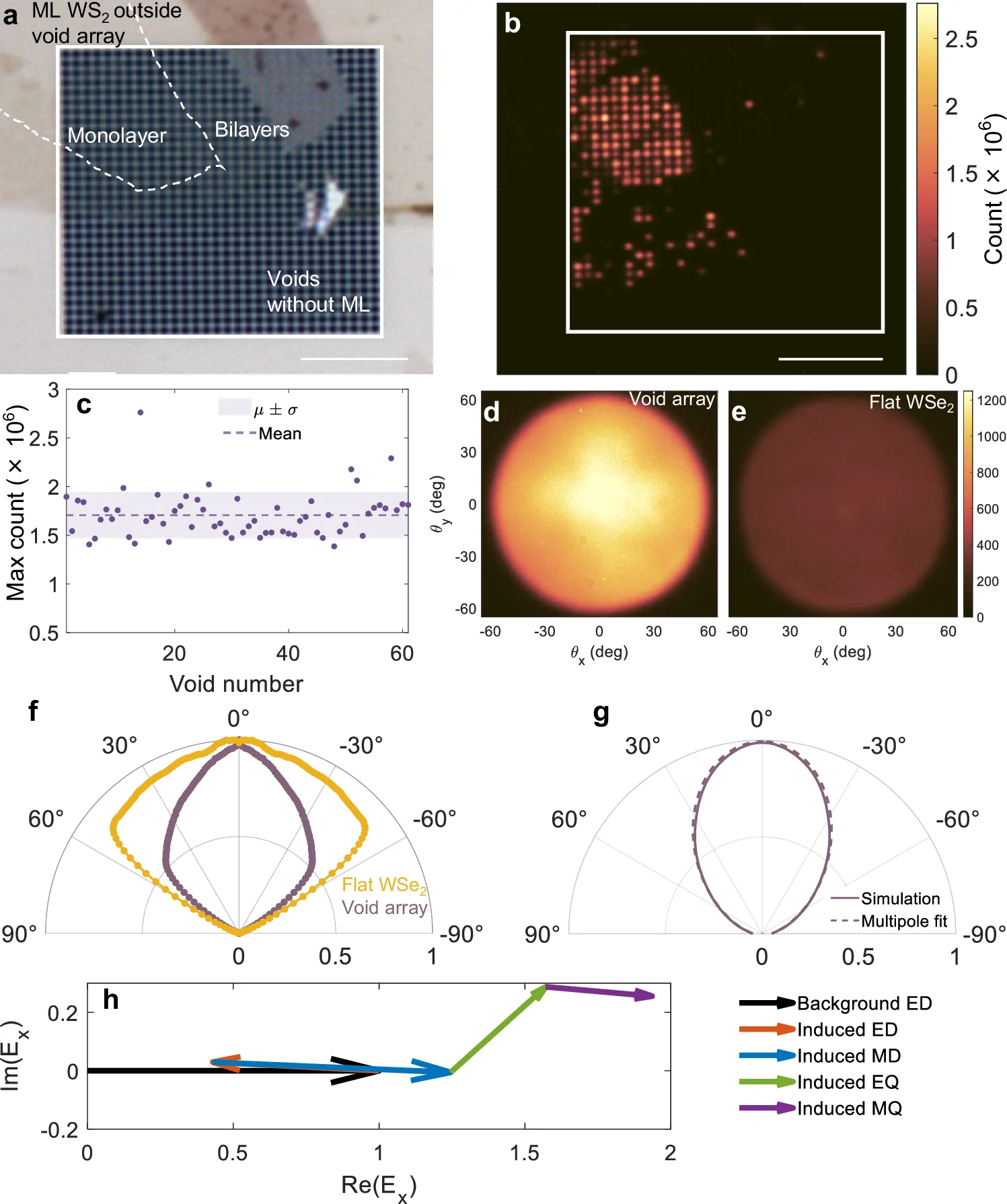
Void-enabled directional emission. **a** Optical image of a WS_2_ monolayer (white dashed line) on a void array. Scale bar: 5 μm. **b** PL map of the same region, showing brighter emission from monolayer-on-void regions. Scale bar: 5 μm. **c** Maximum PL count of individual voids covered by monolayer WS_2_. The black dashed line indicates the sample mean (*μ*) of the maximum PL intensity, and the shaded band shows the standard deviation (±*σ*) around the mean. **d** Back-focal-plane image of a monolayer-on-array configuration. **e** Back-focal-plane image of a monolayer-on-flat WSe_2_ configuration. **f** Azimuthally averaged one-dimensional angular PL profiles (normalized), comparing the void-array emission with the flat WSe_2_ reference. **g** Simulated (solid) and multipole-reconstructed (dashed) far-field emission pattern for a void of dimensions *h* = 330 nm and *d* = 400 nm. **h** Phasor representation of the total electric far-field in the forward emission direction decomposed into different multipolar contributions.

A key contribution to the enhanced PL emission arises from the ability of Mie voids to direct the emitted light toward the collection objective. To investigate this directionality, we perform angle-resolved back-focal-plane imaging under wide-field illumination (see “Methods”). Because the collection area of the optical setup (≈30 μm^2^, see Supplementary Section 10, Fig. S10) is much larger than the area of a single void (≈0.2 μm^2^), measurements on individual voids are not feasible. We therefore fabricated void arrays with a 500 nm pitch and designed the void resonance wavelength to overlap spectrally with the WS_2_ monolayer exciton wavelength (see Supplementary Section 11 and Fig. S11). This ensures that the collected signal predominantly originates from void-coupled regions. Fig. 5a shows an optical micrograph of a WS_2_ monolayer placed on such a void array (with *h* = 336 nm and *d* = 406 nm), and the corresponding PL map in Fig. 5b confirms enhanced emission localized above individual voids. The peak PL intensity shows only minor variations from void to void (see “Methods”), indicating uniform emission across the array (Fig. 5c).

Figures 5d,e display the raw back-focal-plane PL images from a WS_2_ monolayer on a void array and on flat WSe_2_, respectively. The axes (*θ*_*x*_, *θ*_*y*_) correspond to collection angles along the two lateral directions defined by the NA of the objective lens (NA = 0.90). The void array produces a bright central lobe whose intensity decreases toward the periphery, indicating that emission near the surface normal (*θ* ≈ 0^∘^) dominates. In contrast, the flat WSe_2_ reference exhibits an almost angle-independent, Lambertian-like distribution. Because the azimuthal variations in Fig. 5d,e are relatively minor, we approximate the emission as radially symmetric and average the intensity over the azimuthal angle to obtain a one-dimensional polar intensity profile (using a similar process as described in Supplementary Section 7).

To compare the void-coupled and planar emission, we account for the planar regions between neighboring voids that do not contribute to directionality. This is done by an area-weighted subtraction based on the planar fill factor, corresponding to the fraction of planar surface within the array period. The subtraction assumes that PL from different regions adds incoherently, which is appropriate for spontaneous emission. A detailed description of this procedure and its limited impact on the overall result are provided in Supplementary Section 11 and Fig. S11.

The resulting normalized angular profiles are shown in Fig. 5f. The void-coupled emission at normal incidence is approximately 83% higher than that at an angle of 50^∘^, corresponding to a forward-to-reference directionality of about 2.6 dB. For the flat WSe_2_ reference, the emission changes by less than 10% over the same angular range. The pronounced central beaming observed for the void arrays thus provides clear experimental evidence that Mie voids efficiently channel emission into the forward direction.

To gain further insight into the physical origin of this directional emission, we simulate the far-field radiation pattern of a dipole-excited isolated void with dimensions *h* = 330 nm and *d* = 400 nm, matching the experimental void dimensions, and perform a Cartesian multipole decomposition of the induced far-field (see “Methods”). As shown in Fig. 5g, the multipole-expanded field faithfully reproduces the simulated angular distribution, confirming that the radiation can be captured by a finite set of electric and magnetic dipole (MD) and quadrupole contributions. The agreement between simulation and reconstruction demonstrates that the forward-directed emission arises from the coherent superposition of void-supported multipoles, which are excited by the ED.

The interference mechanism underlying this directionality can be elucidated through a phasor representation of the electric field evaluated along the forward direction (Fig. 5h). The phasor diagram shows the relative amplitudes and phases of the individual multipolar contributions and illustrates how they interfere to produce the observed directional emission. The field contributions from the background ED and the induced multipoles are represented as complex vectors and summed sequentially. Notably, the induced ED partially opposes the background ED along the forward direction, while the induced MD, the induced electric quadrupole (EQ) and the induced magnetic quadrupole (MQ) contribute constructively. The resulting phasor sum yields an enhanced total field in the forward direction, illustrating that the observed beaming arises from the coherent interference between multiple multipolar contributions. This analysis provides a clear physical picture of how the void structure reshapes the emission pattern through multipole coupling.

## Discussion

We have demonstrated that Mie voids provide a powerful and conceptually distinct route to control light emission at the nanoscale. By coupling an atomically thin WS_2_ monolayer to Mie void resonators formed in WSe_2_, we achieved simultaneous enhancement and directionality of PL within an all-van der Waals architecture. Electromagnetic simulations and optical measurements show that the PL enhancement arises from the synergistic interplay between strong field localization inside the air void and constructive interference between the emitter and the void resonance. Experimentally, this coupling leads to a 600-fold increase in PL intensity and pronounced beaming of light along the surface normal.

The presented two-step fabrication method enables independent control of void diameter and depth, providing a pathway to engineer the optical response of van der Waals materials with nanoscale precision. Because Mie voids relax the need for low-loss dielectric materials, they can be realized in a wide range of high-index semiconductors and combined with diverse quantum emitters. This opens a new design space for manipulating nanoscale emitters. Simulations further indicate that increasing the void depth shifts the field localization deeper inside the cavity, which may be attractive for integrating quantum dots or single molecules placed within the void. Stacking such a configuration with another layered material such as hBN could additionally shield the embedded emitters from the environment. These avenues remain open for exploration and may extend the Mie void platform toward quantum photonics based on hybrid van der Waals architectures.

## Methods

### Fabrication

The device fabrication proceeds through a sequential multi-step process (see Fig. S12). WSe_2_ flakes are mechanically exfoliated using 3M Scotch Magic Tape 810 from bulk WSe_2_ crystals from HQ Graphene onto a Si substrate and inspected to identify flakes matching the desired thickness and surface quality for subsequent void patterning. Then we spin-coat electron-beam resist to define the etch mask. A 4wt% PMMA (996 k) solution in anisole is spun at 2000 rpm for 60 s and baked at 170 ^∘^C for 300 s. Circular mask openings are then patterned by electron-beam lithography using a 30 kV Raith eLINE Plus with a 30 μm aperture and dose of 230 μC/cm^2^. The sample is developed in IPA:H_2_O (3:1, 60 s) followed by an IPA rinse (30 s). Then the voids are fabricated into WSe_2_ by reactive-ion etching. First we use an initial O_2_ plasma descum to remove residual resist, followed by an SF_6_ plasma etch to define the voids. We use two parameter sets: (i) O_2_ plasma clean (9.15 mTorr, 30 W, 30 sccm, 10 s), followed by an SF_6_ etch (9.15 mTorr, 30 W, 30 sccm, 33 s); and (ii) O_2_ plasma descum (10 mTorr, 30 W, 40 sccm, 10 s), followed by an SF_6_ etch (10 mTorr, 30 W, 30 sccm, 150 s). The resist is stripped in acetone and the sample is rinsed sequentially in IPA and DI water, leaving open voids.

The next step is to prepare thick WSe_2_ flakes that serve as the bottom WSe_2_ substrate underneath the resonators. Thick WSe_2_ flakes from bulk crystals (HQ Graphene) are exfoliated using thermal-release tape on a substrate. Applying heat of roughly 100 ^∘^C disengages the tape adhesive, allowing us to transfer pristine flakes (up to a few micrometers thick). The etched WSe_2_ flakes containing the voids are transferred onto such a thick WSe_2_ flake. We assemble the stack using a dry-transfer method with a 10% polycarbonate (PC) film on a polydimethylsiloxane (PDMS) stamp (HQ Graphene transfer system, product code HQ2D MOT). The void-patterned thin flake is picked up at 110 ^∘^C. For placement on the thick WSe_2_ flake, the stage is heated to 180 ^∘^C to melt the PC and release the stack. Residual PC is removed by immersion in chloroform for 10 min.

For large-area monolayers, we employ Au-assisted exfoliation combined with thermal release tape (TRT). Thin flakes are first mechanically exfoliated using Scotch tape (3M Scotch Magic Tape 810). A 50 nm Au film is then thermally evaporated onto the tape under high vacuum (6 × 10^−6^ mbar) using an Oerlikon UNIVEX 250 evaporation chamber. The Au thickness is determined from a deposition rate of 0.3 Å *s*^−1^, and the Au layer serves as an adhesion layer for the subsequent transfer process. The Au-coated exfoliation tape is then laminated onto a thermal release tape such that the flakes remain on top while the Au layer is positioned underneath. This Au/TRT stack is subsequently brought into contact with the target substrate and gently pressed to ensure uniform adhesion of the flakes. The sample is then heated to activate the thermal release tape, allowing clean detachment of the tape while leaving the flakes adhered to the substrate with residual Au. The remaining Au is removed by etching in KI solution (or aqua regia), followed by rinsing in deionized (DI) water. No additional cleaning steps are performed to preserve the pick-up yield. The high surface energy of Au and its uniform adhesion selectively “pull” the outermost TMD layer, enabling millimeter-scale monolayer exfoliation.

The WS_2_ monolayer is encapsulated in hBN and the resulting hBN/WS_2_/hBN stack is transferred onto the void device to complete the heterostructure. We use the same PC/PDMS dry-transfer approach as previously described. Thin TMDC flakes can typically be picked up at 90 ^∘^C to 100 ^∘^C, while for monolayer crystals we find that increasing the temperature to roughly 120 ^∘^C yields more reliable adhesion to PC.

### Photoluminescence measurements

We use a custom-built confocal microscope to record high–resolution PL maps and spectra. Excitation is provided by a 532 nm solid–state laser (Cobolt, Hübner GmbH), whose power is varied via a rotating half–wave plate. Both excitation and collection are performed through a diffraction–limited Nikon TU Plan Fluor 100×, NA = 0.95 EPI D objective. The laser power for the measurement is around 30 μW. Confocal detection is realized by coupling the collected PL into a single–mode optical fiber, and sample scanning is achieved with a three-axis piezoelectric stage. In the detection path, a 550 nm dichroic mirror and a 532 nm notch filter separate excitation light from the emitted PL. PL is recorded using an avalanche photodiode (APD) single-photon detector. To maximize collection efficiency, we first scan the sample position along the z-axis and record the photon counts at different distances between the stage and the objective. We then identify the z-position that yields the highest photon count and perform the PL mapping at this focus (see Fig. S13). For spectrally resolved measurements, the PL output is diverted via a fiber–coupled beamsplitter into a spectrometer equipped with a piezo–cooled CCD camera (Andor-Solis SR-303i, 150 grooves/mm grating with blaze at 800 nm). To determine the PL peak intensity distribution shown in Fig. 5c, we include only those voids whose maximum PL intensity is at least 50% of the peak intensity of the brightest void in the array.

### Angle-resolved photoluminescence measurements and bright-field microscopy

Bright-field micrographs were obtained using a Nikon Eclipse LV100ND microscope under white-light illumination (Thorlabs OSL2). For the PL images we used a CoolLED pE800 LED system with a broadband illumination ranging 525–550 nm. We only collect the emission from the A-exciton using a band-pass filter (Semrock, ranging 590–650 nm) and a dichroic mirror (FF570-Di01).

The back-focal-plane PL imaging setup, consisting of three free-space lenses, was integrated with the above mentioned optical microscope and an Andor Kymera 328i spectrograph to capture angle-resolved images in *k*-space. To obtain the emitted signal we employed the same broadband excitation ranging 525–555 nm mentioned before and collected the emission between 590 and 650 nm with a high-NA (0.90) objective to access the widest angular range. An iris was placed at the intermediate image plane to ensure that we collect emission only from the region of interest, while a relay lens reimaged the back focal plane of the objective onto the spectrograph. This configuration converted the real-space image into its Fourier-plane representation, providing direct access to the angular distribution of the emission. The emission angles were retrieved from the recorded pixel positions using the relation $\sin \theta=(r/{R}_{{{\rm{max}}}})\,{{\rm{NA}}}/n$, where *r* is the radial pixel distance from the optical axis, *R*_max_ is the pupil radius in pixel, NA is the numerical aperture of the objective, and *n* is the refractive index of the collection medium (1 for air). The optical setup is similar to that used in our earlier work^51^.

### Electromagnetic simulations

Three-dimensional electromagnetic simulations are conducted using COMSOL Multiphysics, which solves Maxwell’s equations using the finite element method. The simulation model consists of a WSe_2_-air interface with a cylindrical void etched into the WSe_2_ surface (see Fig. S13). The coordinate system is defined with *z* as the surface normal and (*x*, *y*) as the in-plane coordinates. Both the linear polarization of the incident excitation plane-wave field and the dipole moment of the emitting dipole are along *x* (see Fig. S13). The complex anisotropic refractive index of WSe_2_ is taken from ref. ^52^. Domain terminations were as follows: top and lateral boundaries (for air domain) used perfectly matched layers to absorb outgoing fields, while the air-WSe_2_ interface is modeled using an impedance boundary condition. To evaluate the emission efficiency, the Poynting vector was integrated on a plane with an area dictated by the NA of the objective lens (NA = 0.9), such that only angles $\theta \le \arcsin ({{\rm{NA}}})$ are collected. The integration plane is placed at a vertical distance of 1.5 *μ*m from the void surface, so that the recorded fields are in the far-field regime.

### Multipole decomposition

The electromagnetic response of an isolated cylindrical void is computed using finite-element simulations in COMSOL Multiphysics in the frequency domain at a wavelength of *λ* = 610 nm. The system is excited by an $\hat{{{\bf{x}}}}$-oriented ED point source positioned laterally at the center and vertically at the opening of the void. The surrounding medium is air, and the computational domain is terminated by perfectly matched layers. The cylindrical void in WSe_2_ is modeled using an impedance boundary condition. To perform the multipole decomposition, the electric field is sampled on a hemispherical surface in the air region of radius *R* = 2 μm centered at the dipole position. Simulations are performed both with and without the void structure, allowing extraction of the induced field as the difference between the total and background fields. Only the tangential components of the electric field are retained in order to isolate the far-field contribution.

The induced electric far-field is decomposed into Cartesian multipoles by fitting it to a linear combination of ED, MD, EQ, and MQ radiation patterns. The angular dependence of the induced field is modeled as^53^

$${{\bf{E}}}(\hat{{{\bf{n}}}}) 	 \propto \ \hat{{{\bf{n}}}}\times ({{\bf{p}}}\times \hat{{{\bf{n}}}})+\frac{1}{c}({{\bf{m}}}\times \hat{{{\bf{n}}}})\\ 	+\frac{ik}{c}\,\hat{{{\bf{n}}}}\times \left(\hat{{{\bf{n}}}}\times ({{{\bf{Q}}}}^{e}\cdot \hat{{{\bf{n}}}})\right)+\frac{ik}{6c}\,\hat{{{\bf{n}}}}\times ({{{\bf{Q}}}}^{m}\cdot \hat{{{\bf{n}}}}),$$

where $\hat{{{\bf{n}}}}$ denotes the observation direction, **p** and **m** are the ED and MD moments, and **Q**^*e*^ and **Q**^*m*^ are symmetric, traceless quadrupole tensors. The multipole coefficients are obtained by solving a complex least-squares problem that minimizes the deviation between the simulated tangential field and the multipole expansion evaluated at all sampled directions. The quadrupole tensors are expanded in a basis of symmetric, traceless matrices, reducing the problem to a linear fit with a finite number of unknown coefficients.

## Supplementary information

## Data Availability

The data generated in this study, including the experimental data, numerical simulation data, and processed datasets sufficient to reproduce all plots presented in the main article and Supplementary Information, have been deposited in Zenodo under DOI: 10.5281/zenodo.21250650.
