# Supplementary material for: Enhanced and directional light emission from two-dimensional excitons using Mie voids
Source: Nat Commun. 2026 Aug 5;17:9417. doi: 10.1038/s41467-026-76273-1 (PMC13538506; doi:10.1038/s41467-026-76273-1)
Supplement: Supplementary file 1 [file 41467_2026_76273_MOESM1_ESM.pdf]

Supplementary Information for

Enhanced and directional light emission from two-dimensional excitons using Mie voids

Avishek Sarbajna[1], Ganesh Ghimire[1], Ilia D. Breev[1], Xavier Zambrana-Puyalto[1], Cheng Xiang[1], Alexander Huck[1], Timothy J. Booth[1], Søren Raza[1]
[1]Department of Physics, Technical University of Denmark, Fysikvej, Kongens Lyngby, DK-2800 Denmark.
*Corresponding author: Søren Raza, E-mail: sraz@dtu.dk

1 Enhancement for SiO_2 substrate

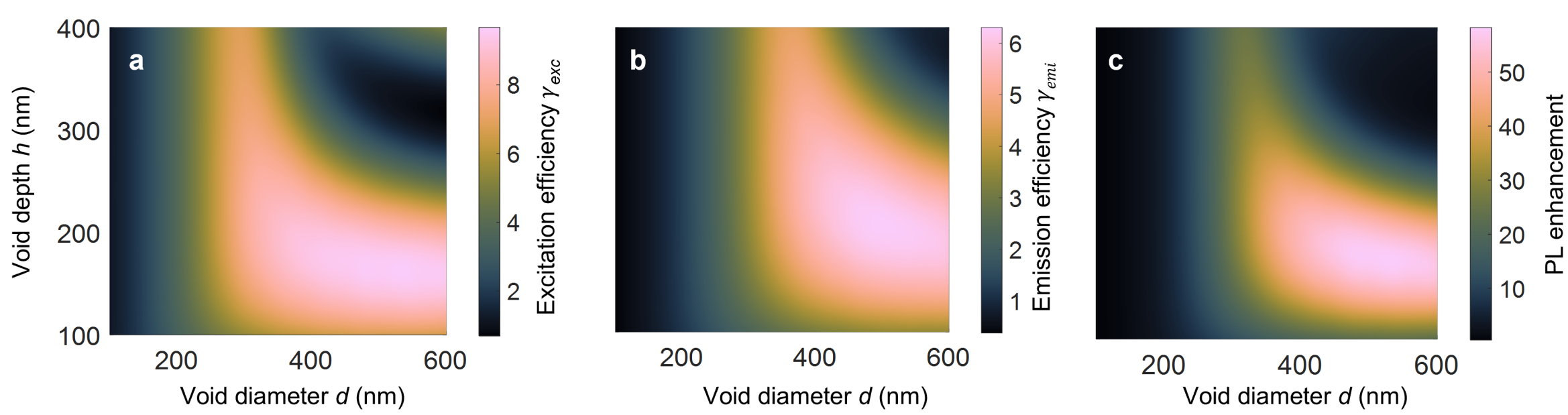

Fig. S1: **Simulated enhancements normalized to SiO_2 substrate. a,** Excitation efficiency γ_{exc}, **b,** emission efficiency γ_{emi} and **c,** resulting PL enhancement as a function of void diameter d and depth h for the dipole-on-void system normalized by dipole on flat semi-infinite SiO_2. The void resonators are still fabricated in bulk WSe_2.

The excitation efficiency, emission efficiency, and the resulting PL enhancement for a dipole placed on WSe_2 Mie voids are normalized to the case of flat SiO_2 substrate in Fig. S1. We observe that the PL enhancement reaches about $60\times$, which is lower than the $1700\times$ enhancement observed when normalizing with flat WSe_2 (shown in Fig. 2 of the main text). The increased PL enhancement relative to flat WSe_2 originates from the quenching nature of the high-index and absorptive properties of WSe_2, which suppresses the excitation and emission processes.

2 Electric field profile inside the void

Enhanced light–matter interaction is achieved when the electric-field maximum spatially overlaps with the excitons in the WS_2 monolayer resting at the void opening. Figure S2 compares the local electric field enhancement of two voids with the same diameter ($d = 540$ nm) but different depths. For a shallow void of depth $h = 160$ nm, the maximal field enhancement occurs at the void opening (Fig. S2a). For a void with increased depth of $h = 300$ nm, electric field maximum shifts inside the void, well below the monolayer position (Fig. S2b). This offset weakens the spatial overlap with the excitons in monolayer WS_2 and thus reduces both the excitation and emission efficiencies. The shallow void with dimensions $(d, h) = (540, 160)$ nm offers the optimal balance between strong confinement and proper alignment with the emitter.

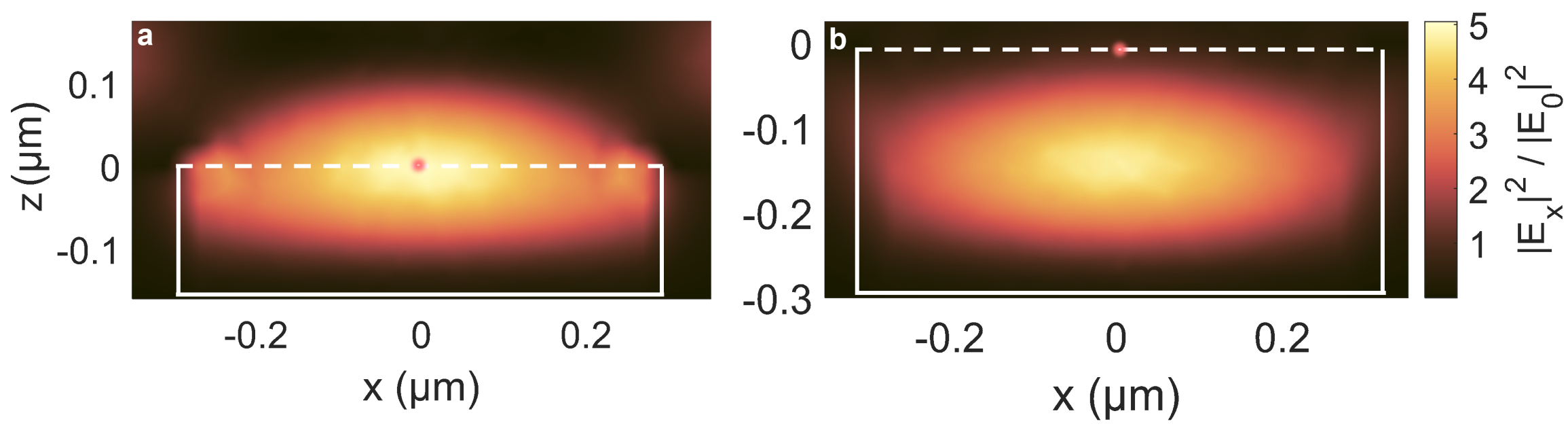

Fig. S2: **Field distribution inside void resonator.** Spatial distribution of the electric field intensity $|E_x|^2/|E_0|^2$ inside two cylindrical voids of identical diameter ($d = 540$ nm). **a,** A shallow void with depth $h = 160$ nm concentrates the field at the void opening. **b,** Increasing the depth to $h = 300$ nm shifts the field maximum inside the void, reducing its overlap with the dipole. White rectangles mark the void cross-sections; pink dots highlight the dipole positions.

3 Interference model for excitation and emission processes

The excitation and emission enhancement processes due to the void resonator can be understood within a single framework based on a one-dimensional constructive-interference condition inside the void. Light that enters the void is approximated as a plane wave that propagates with an effective wavenumber

$$k_z = k_0 \mathrm{Re}(n_{\mathrm{eff}}), \tag{1}$$

where $k_0 = 2\pi/\lambda$ is the free-space wavenumber and n_{eff} is the complex effective index of the fundamental HE_{11} mode of an infinitely-long cylindrical waveguide with an air core and WSe_2 cladding (Fig. S3a). This mode possesses dipolar symmetry [1] and represents the dominant field distribution excited by an in-plane dipole or by an incident plane wave polarized along the void axis (see inset of Fig. S3a). The complex effective index n_{eff} is obtained numerically from a waveguide mode calculation and depends on both the void diameter d and the wavelength λ.

At the bottom of the void, the wave reflects at the air–WSe_2 interface with a reflection coefficient approximated by the normal-incidence Fresnel equation

$$r = \frac{n_{\mathrm{eff}} - n_{\mathrm{WSe_2}}}{n_{\mathrm{eff}} + n_{\mathrm{WSe_2}}}, \tag{2}$$

and corresponding reflection phase $\phi = \arg(r)$. Due to the large index contrast between the effective index and the complex refractive index of WSe_2, the reflection phase is close to π and nearly independent of void diameter (Fig. S3b).

The total field at the void opening results from the superposition of the directly incident wave and the reflected wave that has propagated down and back through the void. Constructive interference at the void opening, and thus maximal electric field amplitude at the emitter position, occurs when

$$2k_z h + \phi = 2\pi q, \tag{3}$$

where h is the void depth and q is an integer. The first constructive-interference maximum occurs for $q = 1$, resulting in a depth-diameter relation given by

$$h(d) = \frac{2\pi - \phi(d)}{2k_0 \mathrm{Re}\left[n_{\mathrm{eff}}(d)\right]} \tag{4}$$

Equation (4) provides a unified description of both excitation and emission enhancements. During excitation (at $\lambda = 532$ nm), maximal electric-field enhancement at the void opening is obtained when Eq. (4) is satisfied. During emission (at $\lambda = 610$ nm), the same phase condition governs both the local field experienced by the emitter (Purcell enhancement) and the interference between the emitter and the void resonance that leads to directional emission. The total forward-directed emission is maximized when the wave that is initially emitted downward into the void, reflected at the bottom, and subsequently radiated upward constructively interferes with the wave that is directly emitted upward. The directionality condition is therefore identical to that for maximal field at the void opening and is given by Eq. (4). The dependence of n_{eff} and ϕ on the void diameter d and wavelength thus predicts the depth-diameter trends observed in both excitation and emission efficiencies (Fig. S3c).

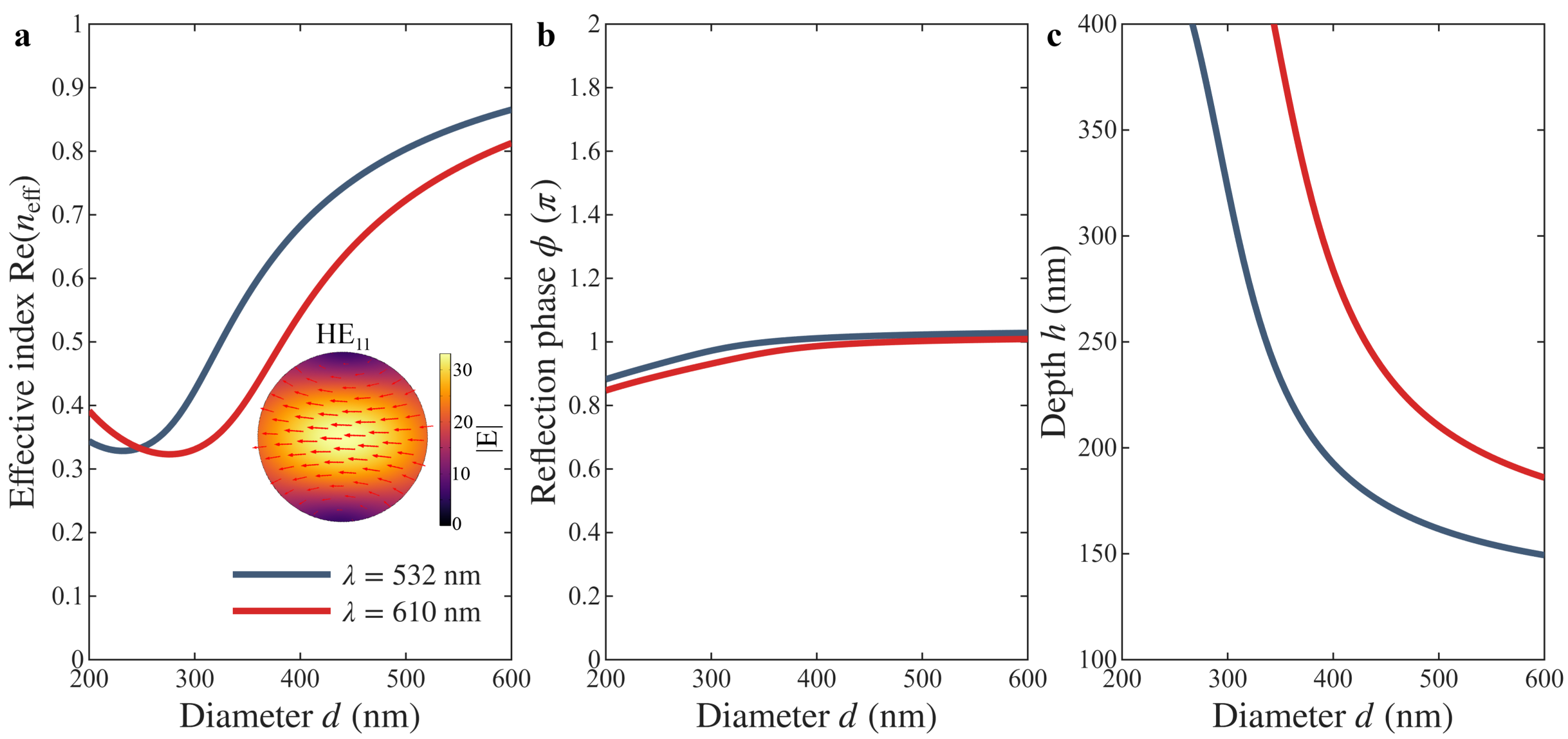

Fig. S3: **Interference model of void resonator**. **a,** Real part of the effective index $\mathrm{Re}(n_{\mathrm{eff}})$ for the fundamental HE_{11} mode of a cylindrical waveguide of diameter d composed of an air core and WSe_2 cladding. Two wavelengths corresponding to the excitation (blue) and emission (red) wavelengths are shown. The inset depicts the electric-field direction (arrows) and magnitude (color) of the dipolar HE_{11} mode for $d = 400$ nm. **b,** Reflection phase ϕ at the effective index-WSe_2 boundary. The phase is close to π and nearly constant for all diameters. **c,** Void depth versus diameter resonance condition, given by Eq. (4), for the excitation (blue) and emission (red) wavelengths. These relations are also displayed in Figure 2 of the main text.

4 Comparison with Si nanodisks

To assess the performance of a dielectric resonator, we simulate the excitation enhancement, emission enhancement, and resulting PL enhancement for a dipole placed on a WSe_2 substrate beneath a Si nanodisk. The dipole position is kept fixed at the WSe_2 surface, while the nanodisk height and diameter are varied to generate two-dimensional parameter maps. In addition, the vertical separation between the substrate and the Si nanodisk is varied to represent the finite spacer (like hBN) thickness between the monolayer emitter and the resonator (Fig. S4a). Figure S4b–d and Fig. S4e–g present two representative cases corresponding to emitter–resonator gaps of 1 and 5 nm, respectively.

Several clear trends emerge from these simulations. First, even for the 1 nm-gap case, the geometries that maximize the excitation enhancement and those that maximize the emission enhancement do not fully coincide (Fig. S4b,c). As a result, the condition for maximum PL enhancement is limited by an incomplete overlap between the excitation and emission optima (Fig. S4d). Importantly, these simulations do not imply that Si nanodisks cannot provide PL enhancement. Rather, they show that high enhancement is achieved only within a relatively narrow region of the geometrical parameter space where the optima for excitation and emission overlap. Second, when the gap is increased to 5 nm, this mismatch becomes more pronounced (Fig. S4e,f). The optimal regions for excitation and emission shift further apart in parameter space, and the resulting PL enhancement shows a clear reduction in the achievable maximum value (Fig. S4g). The Si nanodisk therefore exhibits strong sensitivity to resonator geometry, with only a narrow region of parameter space yielding high PL enhancement.

This sensitivity originates from the spatial distribution of the resonant field. Unlike voids, in the Si nanodisk platform, the strongest optical field is confined predominantly inside the resonator, whereas the emitter is located outside the resonator on the WSe_2 surface. Consequently, even a small increase in the emitter–resonator gap significantly reduces the spatial overlap between the emitter and the localized near field. This lowers the combined excitation and emission efficiencies, leading to a decrease in the PL enhancement.

To quantify the fabrication tolerance of the different resonator platforms, we rank the 30 brightest PL enhancement values obtained from the two-dimensional parameter sweeps in resonator height and diameter. Since both parameters were varied using a 20 nm step size, these top 30 values correspond to the 30 grid points in the (h, w) parameter space that yield the highest simulated PL enhancement. For each platform, the ranked PL enhancement values are normalized to the brightest value in that same dataset, i.e. $PL_{\mathrm{norm}}(n) = 100 \times PL(n)/PL(1)$. As shown in Fig. S4h, the void resonator exhibits a slower decay across the top 30 ranked values than the Si nanodisk. For the void resonator, the top 30 PL enhancement values lie between 1513 and 1734, with an average of 1636, indicating that high PL enhancement is maintained across a relatively broad region of geometry space. The slow decay demonstrates that many nearby geometries perform similarly well and therefore that the void geometry is more tolerant to fabrication-induced deviations. By contrast, for the Si nanodisk with a 5 nm gap, the top 30 values span from 144 to 411, with an average of 235, and the normalized curve decreases much more rapidly. This steep decay demonstrates a stronger sensitivity to dimensional variations for the Si nanodisk.

The gap dependence of the Si nanoresonator is summarized in Fig. S4i, where the maximum PL enhancement is plotted as a function of emitter-resonator gap from 1 to 5 nm. The maximum PL enhancement decreases monotonically from 1153 at 1 nm gap to 411.1 at 5 nm gap. This trend shows that the Si nanodisk is highly sensitive to emitter-resonator separation.

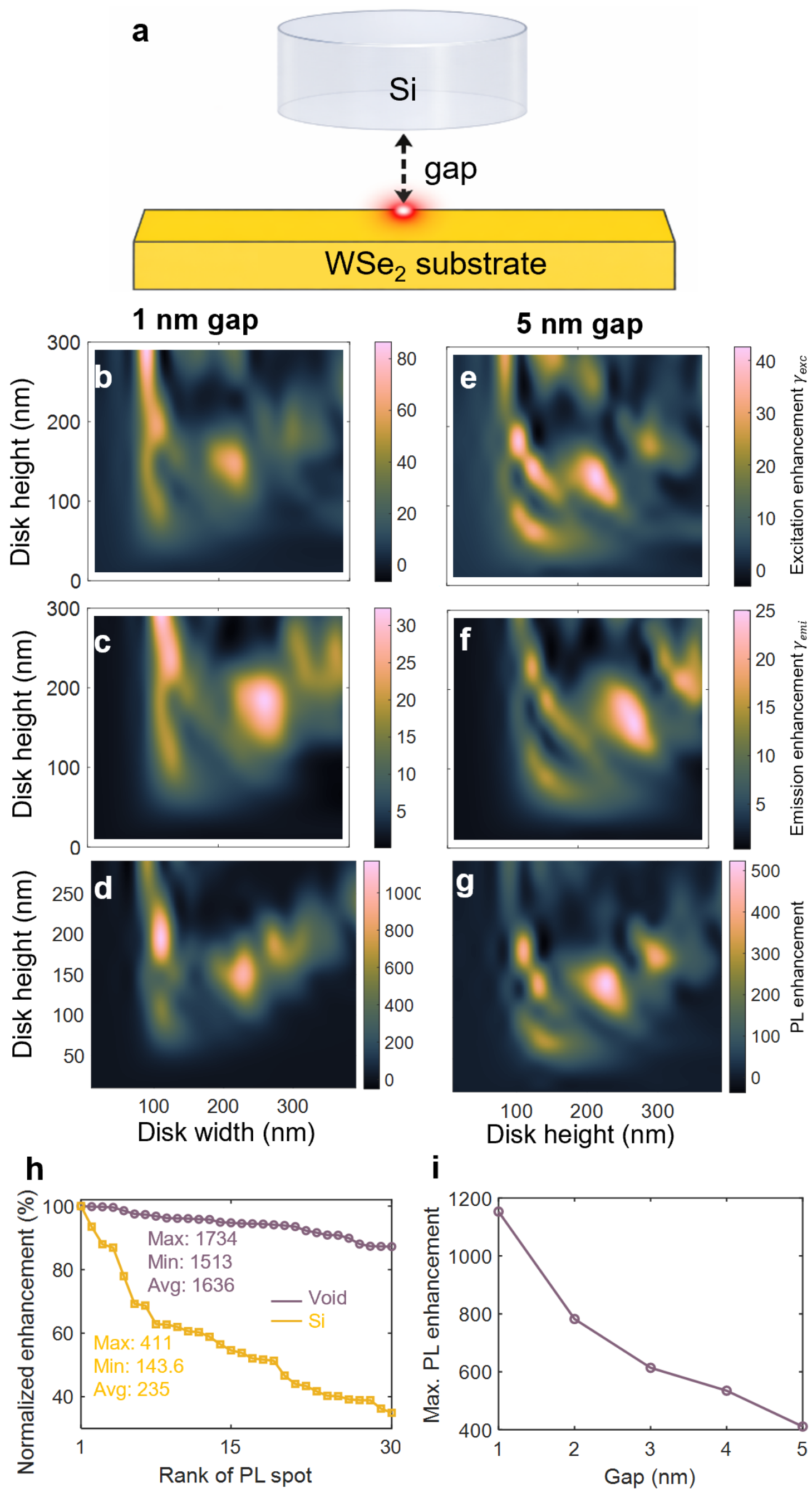

Fig. S4: **Simulated enhancements for Si nanodisk separated from the emitter by a finite air gap.** **a,** Schematic of the simulated geometry. **b–d,** Excitation enhancement, emission enhancement, and PL enhancement for a 1 nm gap. **e–g,** Same as in **b–d**, but for a 5 nm gap. **h,** Normalized PL enhancement of the top 30 brightest parameter combinations for the void resonator and the Si nanoresonator. **i,** Maximum PL enhancement of the Si nanoresonator as a function of emitter–resonator gap.

5 Impact of mask opening and pattern concentration on the etch rate

Etching at the nanoscale depends on multiple factors like feature geometry and pattern density. The size of the mask openings (d_{nom}) control the transport of reactants into the voids and removal of byproducts. Therefore, smaller d_{nom} typically etch shallower for the same etching time. In arrays, microloading further impacts the outcome. In dense arrays (small pitch), neighboring openings draw from the same local flux of active etchants, so reactants are depleted near each void and the vertical etch rate drops. We show the dependence of the void profile on pitch length at fixed etch time using AFM line cuts (Fig. S5). For each of the three nominal diameters, we compare arrays patterned with two different pitches: $500\ \mathrm{nm}$ (red) and $600\ \mathrm{nm}$ (blue). The larger pitch (lower void density) systematically yields deeper and broader profiles, while the smaller pitch exhibits less wide and shallower etching. The surface is at $h = 0$ nm, hence negative values indicate etch depth.

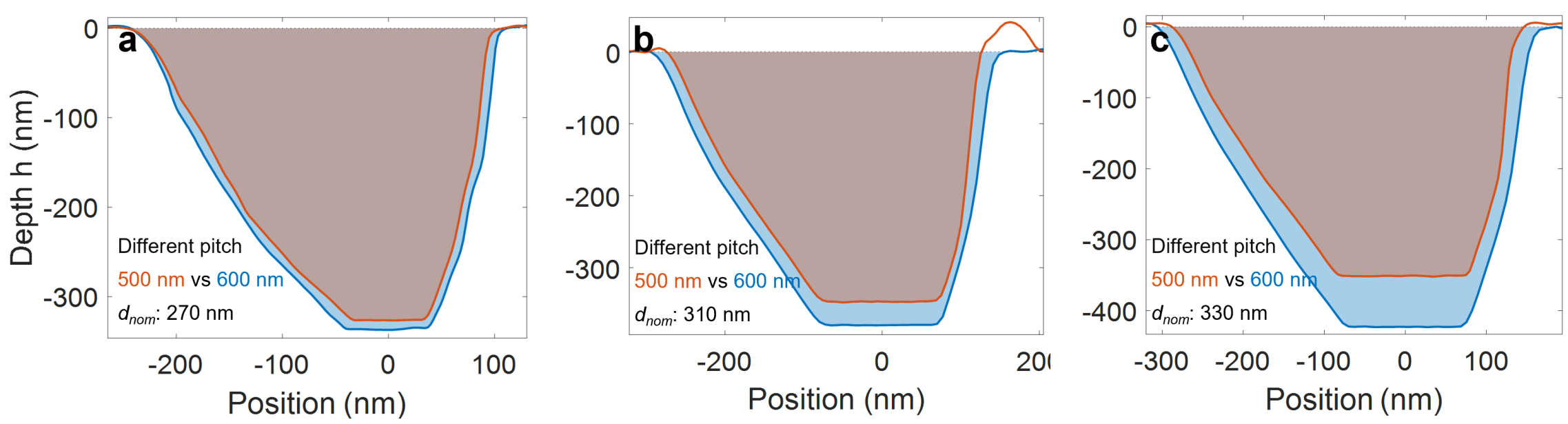

Fig. S5: **Impact of void concentration on etch rate. a-c,** Impact of void concentration. Atomic-force microscopy line cuts of void arrays, comparing voids fabricated with same d_{nom} and etch time but different pitch lengths. Greater pitch length (low concentration) leads to deeper and wider etching.

6 Effect of dipole position on excitation and emission efficiencies

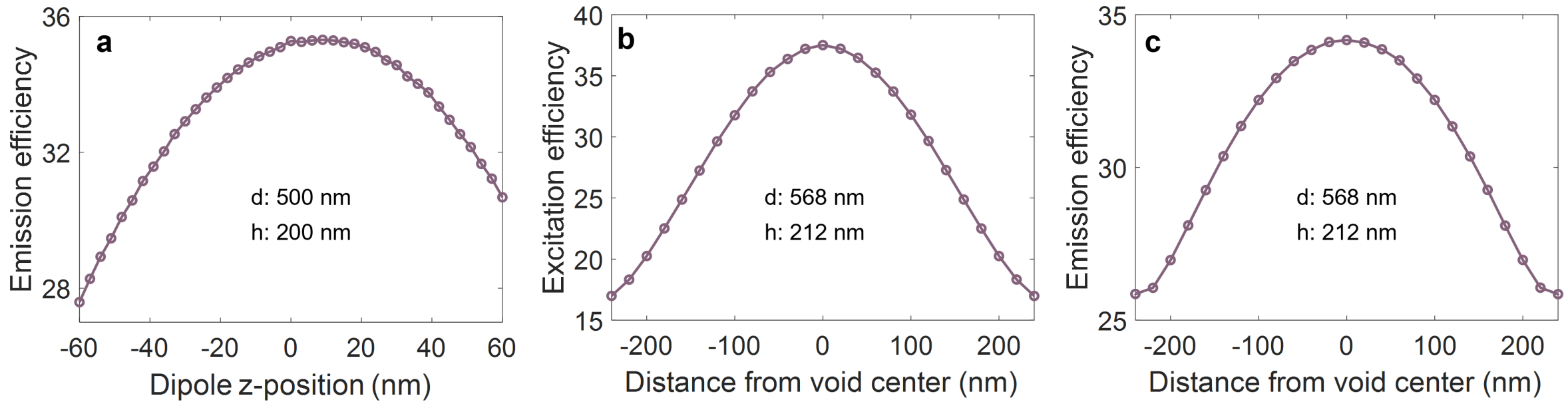

Fig. S6: **Dependence of excitation and emission efficiencies on dipole position. a,** Emission efficiency as a function of dipole position in the out-of-plane (z) direction. The in-plane position is kept constant at the void center. The void dimensions are $(d, h) = (500, 200)$ nm. **b,c** Simulated excitation efficiency and emission efficiencies as the dipole position is translated along the in-plane x-axis at the monolayer plane ($z = 0$). Both curves peak at the void center and decay symmetrically; however, the emission efficiency decays more slowly toward the void rim.

To understand how the vertical position of the emitter influences its emission efficiency, we conduct additional simulations where we translate the dipole along the out-of-plane (z) direction for the case of a void with dimensions $(d, h) = (500, 200)$ nm (Fig. S6a). The in-plane coordinates are fixed to the void center. The efficiency peaks around $z = 0$ nm, corresponding to the top rim of the void and remains nearly constant for dipole displacements up to 20 nm above the void opening. These findings establish the void opening as the optimal emission plane, yet they also reveal a generous fabrication tolerance. Vertical offsets, such as those introduced by encapsulating the monolayer with a thin hBN spacer, have only a minor impact on the emission efficiency.

Fig. S6b-c isolates the two ingredients that determine the spatially-resolved PL enhancement shown in Fig. 4 of the main text. Fig. S6b,c show the excitation and emission efficiencies, as the dipole is translated laterally along the x-axis in the $z = 0$ plane. The void dimensions are $(d, h) = (568, 212)$ nm, matching the sample dimensions shown in Fig. 4d of the main text. Both curves peak at the void center. As the dipole is translated away from the void center, the excitation and emission efficiencies decrease. Near the center their magnitudes are comparable, whereas closer to the rim, the emission efficiency remains noticeably higher than the excitation efficiency.

7 Angular averaging process

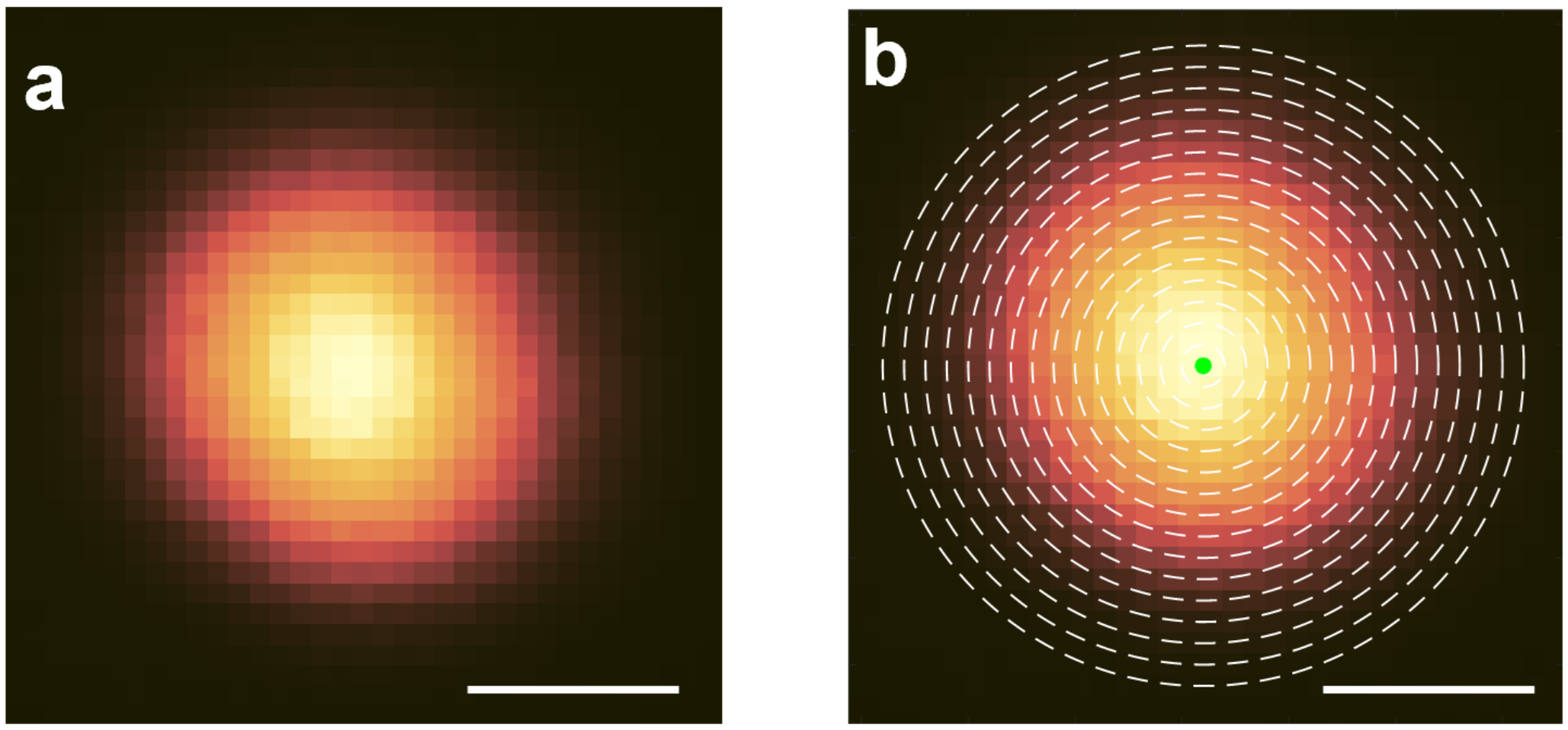

Fig. S7: **Angular averaging of 2D maps into 1D profiles. a,** Example of high-resolution PL emission map of monolayer WS_2 on a void resonator. **b,** Same PL as in **a** with centroid and concentric annuli used for angular averaging. Scale bars: 300 nm

Figure S7a shows a high-resolution PL map of a monolayer-on-void structure. To convert the two–dimensional PL map into the one–dimensional radial profile, we follow the following workflow. First, for each PL map we locate its local maximum intensity (green dot in Fig. S7b) and treat that pixel as the center of the corresponding void. Next, a circular region of interest (ROI) with a radius of 26 pixels (diameter consisting of 51 pixels) is extracted around this center so that the void is exactly in the middle of the ROI. Each pixel corresponds to 30 nm set by the piezo-stage step size. Within the ROI we then compute the mean intensity of one-pixel-wide concentric annuli (white circles in Fig. S7b), stepping radially from 0 to $+25$ pixels. This procedure yields a 26-point vector that captures how the emission decays with distance from the void center. Finally, when we plot the resulting radial trace, it peaks at the void center ($x = 0$) and falls off symmetrically toward the rim.

8 Effect of local strain at the void-monolayer interface

We have performed control experiments to assess whether strain in WS_2 impacts the observed PL enhancement. Figure S8a shows a two-dimensional schematic of the three stacking configurations realized on the same void platform, where the gray region represents the void array. The three cases correspond to a monolayer supported by bottom hBN only, a monolayer covered by top hBN only, and a monolayer fully encapsulated between top and bottom hBN. Figure S8b shows the optical image of the void arrays with different dimensions and identifies the spatial regions corresponding to the different stacking configurations. Figure S8c presents the PL maps measured from these regions superimposed on the bright-field image of the void arrays. Figure S8d shows AFM line cuts comparing their topographies.

The AFM data indicate that the different stacking configurations produce different local strain conditions in the monolayer. For the fully encapsulated case and the bottom-hBN-supported case (case 1 and 3) we do not observe periodic strain modulation induced by the voids. In contrast, when only top hBN is present (case 2), a periodic strain modulation of up to 0.16% is observed, estimated using $\varepsilon \approx \frac{2}{3}\left(\frac{\delta}{r}\right)^2$, where r is the void radius and δ is the maximum deflection of the monolayer at the void center.

Such strain levels are relatively small, and prior studies indicate that significantly larger strain ($> 0.5\%$) is required to produce pronounced PL enhancement in TMDC monolayers [2, 3, 4]. To directly assess the impact on our system, we analyze a high-resolution PL map of the upper void array in Fig. S8e, where seven circular regions of identical area are selected across different parts of the array, spanning the different stacking configurations. For each region, the integrated PL intensity is plotted in Fig. S8f. The total PL intensity remains within a similar range across all regions, with a mean value of 1.9×10^9 counts and a relative standard deviation of 9.7%.

These results show that variations in local strain do not lead to significant changes in the PL intensity, indicating that strain is not the dominant factor governing the observed enhancement. Instead, the enhancement is primarily determined by coupling of the excitons to the Mie void resonators.

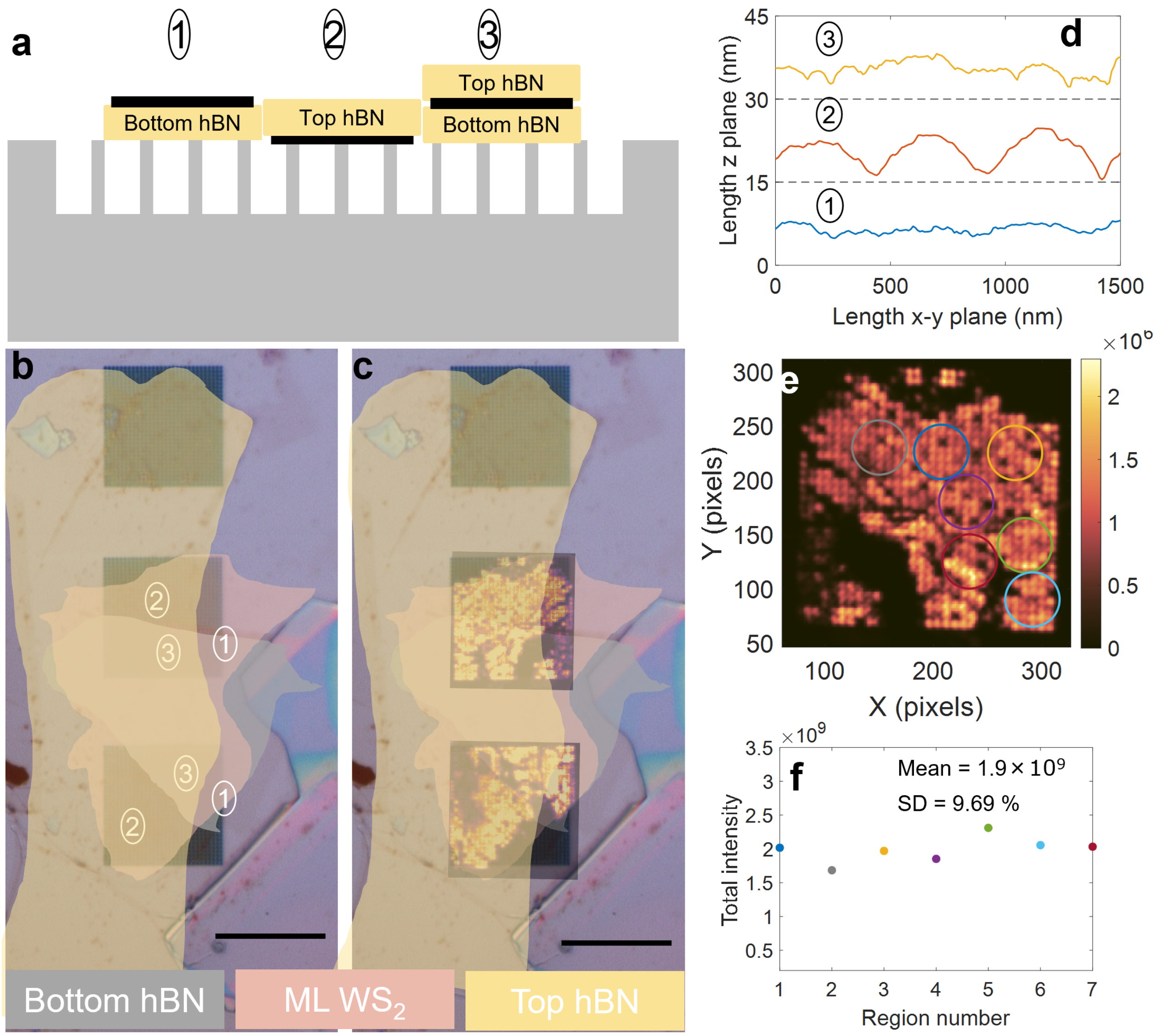

Fig. S8: **Photoluminescence mapping comparing different hBN stacking configurations on the void array device. a,** Two-dimensional schematic of three local stacking configurations on the same void platform, where the gray patterned region represents the void array: (1) monolayer on bottom hBN only, (2) monolayer with top hBN only, and (3) monolayer fully encapsulated by top and bottom hBN. **b,** Schematics on the optical image showing the different flake regions and the corresponding stacking configurations on the device. **c,** PL maps measured from the monolayer on two void arrays. **d,** An AFM line-cut measured along a void array row comparing the topography of the three stacking cases. **e,** Finer PL map of the top void array shown in Figure S8c. Seven circular regions of equal areas were selected across different parts of the array for quantitative comparison. **f,** Total PL intensity extracted from each circular region. Colors of the data points indicate the corresponding circular region in Figure S8e. Scale bar: 5 μm.

9 Finite-spot-size averaging of the simulated spatial PL profile by Gaussian convolution

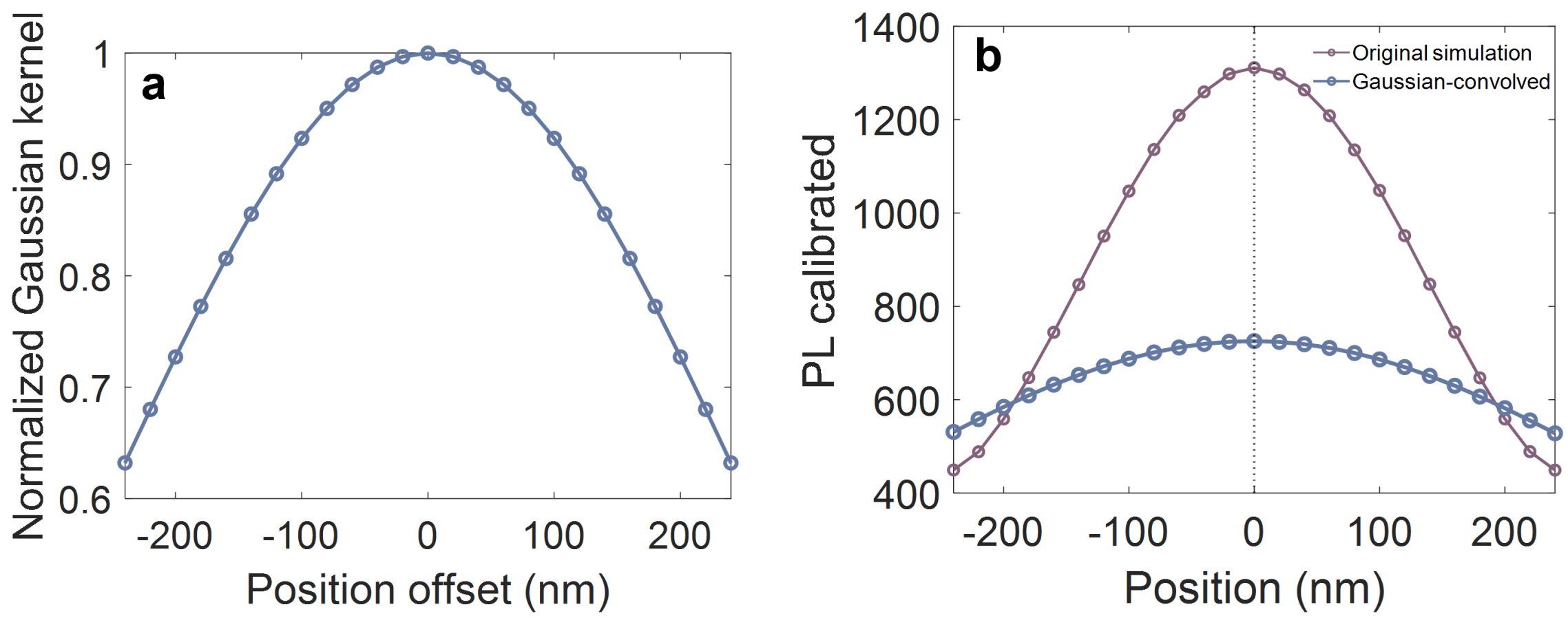

Fig. S9: **Finite-spot averaging of the simulated PL profile by Gaussian convolution. a,** Normalized Gaussian kernel used to model the spatial profile of the excitation spot, plotted as a function of position offset from the void center. **b,** Simulated calibrated PL profile across the void before and after convolution with the Gaussian kernel. The original point-dipole simulation exhibits a peak enhancement of about $1300\times$ at the void center, whereas the Gaussian-convolved profile is broadened and reduced in amplitude, yielding a maximum enhancement of approximately $730\times$ at the center of the void resonator. The vertical dotted line marks the void center.

To account for the finite spatial extent of the excitation spot in the experiment, we convolved the simulated spatial PL profile with a Gaussian kernel corresponding to the measured spot size. For an illumination spot diameter of $1\ \mu$m, we approximate the spatial profile by a Gaussian with a full width at half maximum (FWHM) of $0.59\ \mu$m, corresponding to a standard deviation

$$\sigma = \frac{\text{FWHM}}{2\sqrt{2\ln 2}} \approx \frac{0.59\ \mu\text{m}}{2.355} \approx 0.25\ \mu\text{m}.$$

The normalized Gaussian kernel is therefore written as

$$G(x) = \exp\left\{\left(-\frac{x^2}{2\sigma^2}\right)\right\}, \qquad \widetilde{G}(x) = \frac{G(x)}{\int G(x)\,\mathrm{d}x},$$

and the finite-spot-averaged PL profile is obtained by convolution of the simulated point-dipole PL response $PL_{\text{sim}}(x)$ with the Gaussian kernel,

$$PL_{\text{conv}}(x_0) = \int PL_{\text{sim}}(x)\,\widetilde{G}(x - x_0)\,\mathrm{d}x.$$

Figure S9a shows the normalized Gaussian kernel used for the averaging procedure, while Fig. S9b compares the original simulated PL profile with the Gaussian-convolved result. The point-dipole simulation

predicts a maximum PL enhancement of about $1300\times$ at the void center, whereas the finite-spot averaging reduces the enhancement to approximately $730\times$, much closer to the experimental value. This analysis shows that the experimentally relevant PL enhancement is significantly lower than the ideal point-dipole value because the illumination spot excites not only the void center but also the surrounding region, thereby spatially averaging the response.

10 Area calculation by image processing

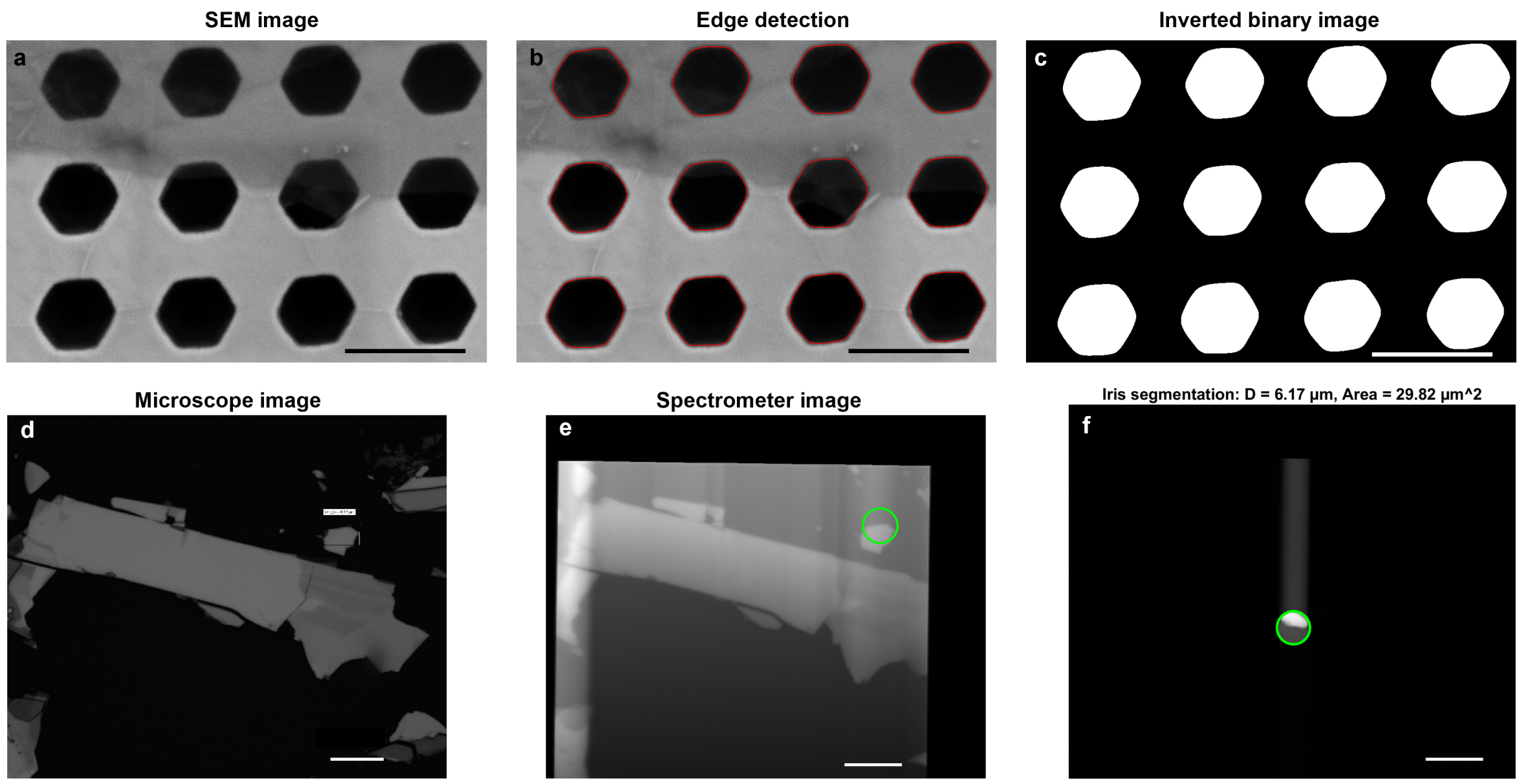

Fig. S10: **Area calculation by image processing. a-c,** SEM image processing: raw SEM image **a,** edge detection **b,** and inverted binary mask **c** used to extract individual void areas and array fill factor. Scale bars: 500 nm. **d-f,** Iris calibration: microscope reference image **d,** spectrometer reference image for pixel-size calibration.**e,** spectrometer image to determine iris diameter and area **f**. The green circle shows the area on the sample from where light is collected through the iris. Scale bars: 10 μm.

Void area analysis from SEM

To extract the area and thereby the diameter of the fabricated voids, we process scanning electron microscopy (SEM) images of the arrays (Fig. S10a). The raw SEM frames are subjected to edge detection to highlight the polygonal void boundaries (Fig. S10b). The detected rims are then converted into a binary mask, inverted to isolate the void interiors, and cleaned of small artifacts (Fig. S10c). Individual void areas and average void area are calculated in real units using the SEM pixel calibration. Once the void area is known, we calculate the fill factor (planar area fraction in the total array area) for the array. The voids are hexagonal in shape because of the Wulff constructions, which leads to a difference in lateral etch rates [5]. Nevertheless, the void resonances are governed mainly by the void volume, and circular and hexagonal voids of comparable volume exhibit similar optical responses [6].

Iris area calculation

A second workflow is used to determine the effective iris aperture by comparing the spectrometer and microscope images. This helps us to determine the required monolayer-on-void array size so that it

covers the entire collection area for the angle-resolved measurements. First, an optical microscope image is acquired as a reference (Fig. S10d). Using this reference, the real-space pixel size is calibrated by measuring the length of a 10 µm scale bar and a 6.96 µm test object. The wide-field spectrometer image is then registered to the microscope frame by selecting corresponding control points, yielding an aligned spectrometer map (Fig. S10e). Once registered, we identify the iris area on a third spectrometer image where the light is only collected through the iris (Fig. S10f) from the area enclosed by the green circle in Fig. S10e. From there the iris diameter and area are calculated.

11 Directional emission from monolayer on void array

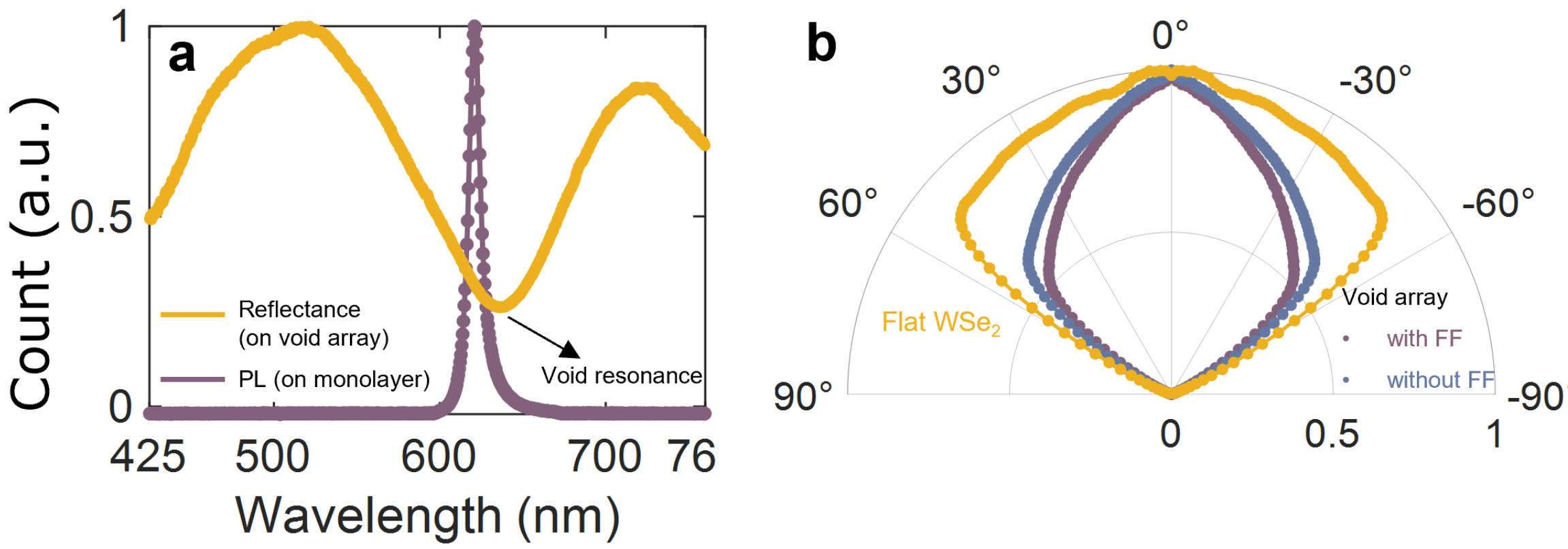

Fig. S11: **Directional emission.** **a,** Normalized reflectance from the void array without monolayer WS_2 (yellow) and PL from the monolayer-on-void array device (purple). The PL emission wavelength matches spectrally with the void resoannce wavelength. **b,** Azimuthal-averaged 1D angular PL emission plots calculated from the back-focal-plane images in Fig. 5d,e of the main text, comparing the case of flat WSe_2 (yellow) to the void array with (purple) and without (blue) fill factor (FF) correction.

Fig. S11a presents the normalized reflectance measurement on the void array and the PL from the monolayer-on-void array, showing that the void resonance (reflectance dip) overlaps spectrally with the PL peak wavelength.

The SEM image of the array (Fig. S10) reveals substantial planar regions between neighboring voids that only weakly contribute to the directionality or PL enhancement. Therefore, we remove the planar contribution from the angularly-averaged PL emission (shown in Fig. 5f of the main text) by using the planar fill factor $FF = 0.54$ derived from the SEM image:

$$I_{\text{void}}(\theta) = I_{\text{array}}(\theta) - FF\, I_{\text{flat}}(\theta),$$

where $I_{\text{array}}(\theta)$ is the measured back-focal-plane intensity of the monolayer-on-void array, $I_{\text{flat}}(\theta)$ is that of the monolayer on flat WSe_2, and $I_{\text{void}}(\theta)$ is the isolated void response. Fig. S11b presents the normalized 1D polar plots comparing emissions with and without the fill factor correction.

12 Supplementary figures related to the Methods section

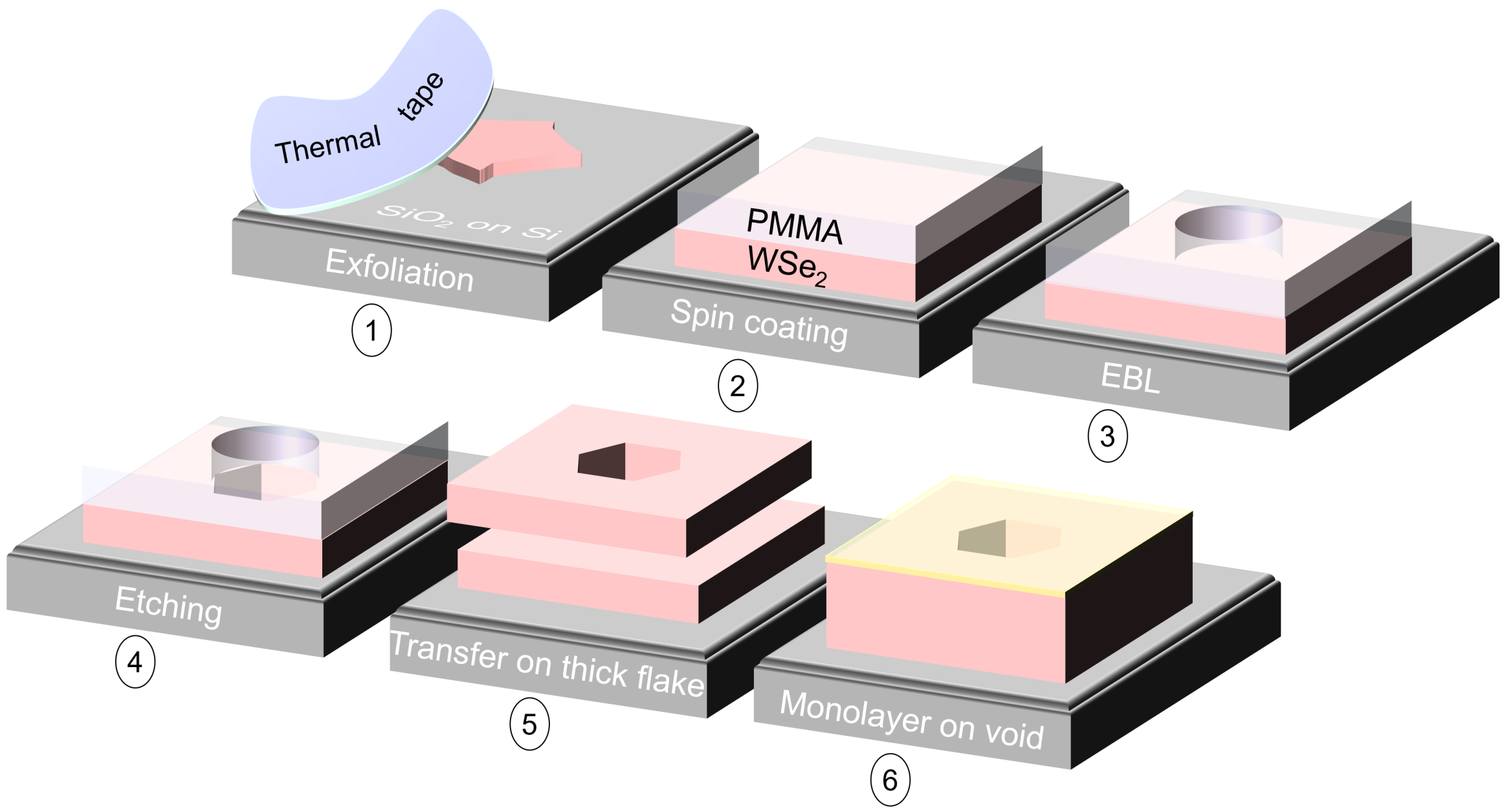

Fig. S12: **Fabrication process.** Step 1: Exfoliation and flake selection. Thin WSe_2 flakes are mechanically exfoliated from bulk crystals onto a clean substrate and inspected to identify flakes with the target thickness and minimal surface roughness for subsequent lithography and etching. Step 2: Spin-coating of PMMA. The selected thin-film WSe_2 flake is coated with PMMA to define an etch mask. Step 3: EBL patterning of the void mask. Circular void openings are written into the PMMA using electron-beam lithography. The exposure pattern can define either isolated voids or periodic arrays. After development, the exposed regions are removed, leaving well-defined openings in the resist that expose the underlying WSe_2 and set the lateral geometry of the subsequent etched voids. Step 4: Reactive-ion etching. The resist pattern is transferred into the thin WSe_2 by dry etching. Once the etching is done, the resist mask is removed from the sample. Step 5: Transferring onto thick flake. The flakes with the fabricated voids are then transferred on a thick WSe_2 flake. Step 6: Transfer of the hBN-encapsulated WS_2 emitter stack. A WS_2 monolayer is encapsulated between thin hBN flakes to protect the monolayer from the environment. The hBN/WS_2/hBN stack is aligned and transferred onto the void device, positioning the emitter region above the void resonators to complete the heterostructure and enable coupling of WS_2 excitonic emission to the void resonances.

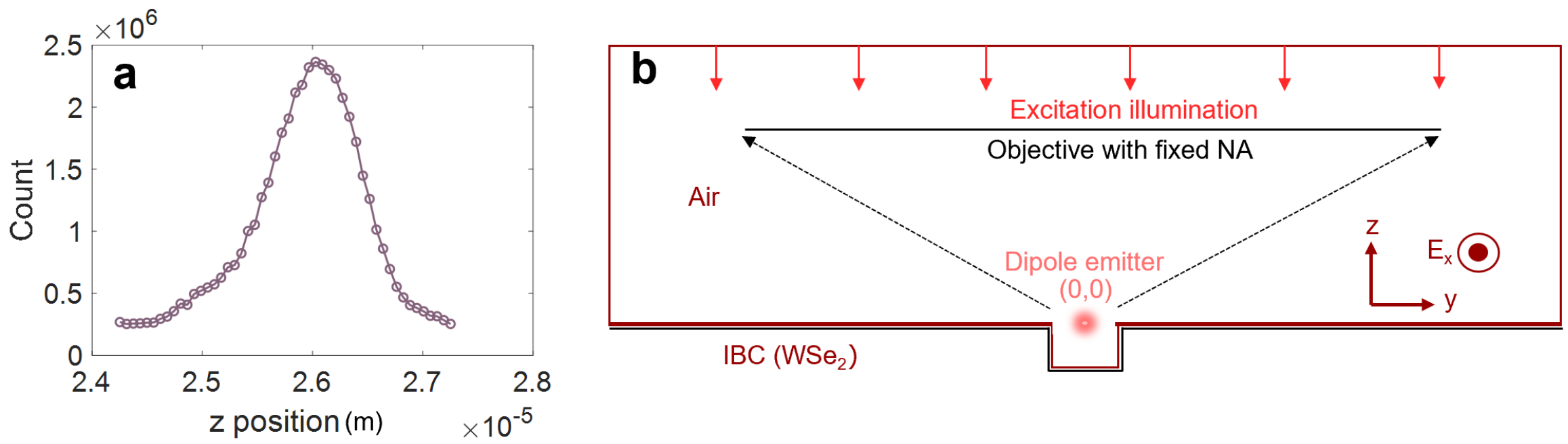

Fig. S13: **Focus optimization and simulation model. a,** Photon counts recorded while scanning the sample position along the optical axis (z) to maximize collection efficiency. The z position at the peak count is chosen as the focus for subsequent PL mapping. **b,** Cross-sectional schematic of 3D simulation model in COMSOL, showing a WSe_2-air interface with a cylindrical void etched into the WSe_2 surface. The exciton emission is modeled by an electric dipole located at $(x, y, z) = (0, 0, 0)$ with a dipole moment along x. The excitation illumination is modeled by a normally-incident plane-wave linearly polarized along x. Outgoing fields are absorbed using perfectly matched layers (PMLs) on the top and lateral boundaries of the air domain. To emulate a semi-infinite WSe_2 substrate without explicitly meshing a thick WSe_2 slab, the bottom boundary is implemented as an impedance boundary condition (IBC) matched to the complex wave impedance of bulk WSe_2 (using the material's complex and anisotropic refractive index). This IBC is appropriate when the substrate can be treated as effectively semi-infinite compared to the penetration depth. Using the IBC significantly reduces the simulation volume and number of degrees of freedom, thereby lowering memory usage and computation time relative to modeling an excessively thick substrate. The black line in the air region denotes the far-field sampling plane used to evaluate the emission efficiency within the numerical aperture of objective lens. It is placed several free-space wavelengths above the void opening (1.5 µm for emission at 610 nm), so that the recorded fields are in the far-field regime. The area of the integration plane is determined by the distance between the plane and the void surface and the NA of the objective lens (0.9).

Supplementary References

[1] Constantine A. Balanis. *Advanced Engineering Electromagnetics*. Wiley, 2 edition, 2012.

[2] Zhiwei Peng, Xiaolin Chen, Yulong Fan, David J. Srolovitz, and Dangyuan Lei. Strain engineering of 2d semiconductors and graphene: from strain fields to band-structure tuning and photonic applications. *Light: Science & Applications*, 9(1):190, 2020.

[3] Riccardo Frisenda, Matthias Drüppel, Robert Schmidt, Steffen Michaelis de Vasconcellos, David Perez de Lara, Rudolf Bratschitsch, Michael Rohlfing, and Andrés Castellanos-Gomez. Biaxial strain tuning of the optical properties of single-layer transition metal dichalcogenides. *npj 2D Materials and Applications*, 1(1):10, 2017.

[4] Shrawan Roy, Xiaodong Yang, and Jie Gao. Biaxial strain tuned upconversion photoluminescence of monolayer WS_2. *Scientific Reports*, 14(1):3860, 2024.

[5] Dorte R. Danielsen, Anton Lyksborg-Andersen, Kirstine E. S. Nielsen, Bjarke S. Jessen, Timothy J. Booth, Manh-Ha Doan, Yingqiu Zhou, Peter Bøggild, and Lene Gammelgaard. Super-resolution nanolithography of two-dimensional materials by anisotropic etching. *ACS Appl. Mater. Interfaces*, 13:41886, 2021.

[6] Avishek Sarbajna, Dorte Rubæk Danielsen, Laura Nevenka Casses, Nicolas Stenger, Peter Bøggild, and Søren Raza. Encapsulated void resonators in Van der Waals heterostructures. *Laser Photonics Rev.*, 19:2401215, 2024.
