# Supplementary material for: Enhanced and directional light emission from two-dimensional excitons using Mie voids
Source: Nat Commun. 2026 Aug 5;17:9417. doi: 10.1038/s41467-026-76273-1 (PMC13538506; doi:10.1038/s41467-026-76273-1)
Supplement: Supplementary file 2 [file 41467_2026_76273_MOESM2_ESM.pdf]

nature portfolio

Peer Review File

Enhanced and directional light emission from two-dimensional excitons using Mie voids

Corresponding Author: Professor Søren Raza

This file contains all reviewer reports in order by version, followed by all author rebuttals in order by version.

Version 1:

Reviewer comments:

Reviewer #1

(Remarks to the Author)
The authors present a nanophotonic platform that utilizes Mie voids to solve a persistent challenge in dielectric nanoantennas: the fact that maximum electromagnetic fields are typically confined into high-index materials rather than accessible at the surface. By inverting the Mie geometry, the authors demonstrate significant photoluminescence (PL) enhancement and directional emission.
The work is innovative and well-written. Overall, the experiments and the presentation are of high quality, and I feel that, in principle, publication would be possible in Nature Communications. However, I have two major points, which I feel need clarification before publication can be considered. Moreover, there are a few important but minor additional questions/comments.
Major points:

1. The PL enhancement is very impressive; however, the observed values do surprise me. Intuitively, I would have expected significantly smaller values, also when comparing to published work on coupling emitters to plasmonic or dielectric systems. Just as a back-of-the-envelope calculation, I would look at the Purcell enhancement factor, which is proportional to lambda^3, Q, and the mode volume, given that the mode volume can be approximated with lambda^3 as well, the enhancement should linearly scale with the quality factor. For the excitation enhancement, I would also roughly assume a factor of Q, as this gives the enhanced fields compared to the far field. Overall, this would be proportional to Q^2, which gives (by looking at Fig 4e, Q approx. 4) a total of about 20. Surely this is just a very rough estimation, but it should give a feeling at least. The reported values are around 600, which is more than an order of magnitude larger and thus difficult for me to understand.
I feel the authors need to make an intuitive case why the enhancement is so much larger as this really collides with my intuition and, as mentioned, other reported values in similar systems
2. One of the key points of the manuscript is the manipulation of the emission properties, which is a physically interesting effect. Again, intuitively, it is evident that the emission pattern should be modified due to the coupling to the void resonance. Measuring back-focal-plane images is a very smart idea for such experiments, however, due to the nature of the experiment, I feel some ambiguities need to be addressed. Most importantly, a model and/or simulation of the radiation pattern is needed. Only such a model, predicting the emission pattern, can convincingly rule out other possible explanations as well as the influences of the experimental challenges, mainly the “normalization” due to the fact that most voids are not covered with the WS2 monolayer. Also, I wonder if there might be phased-array-like effects, which would also modify the emission pattern? Also, I wonder, what limits the sport size? Should a smaller aperture in the beam path not allow a smaller interrogated region?

Additional issues
1. The authors state that roughly 50% loss in predicted enhancement stems from the limited spot size of the diffraction-limited illumination laser. To what extent might other effects contribute to this discrepancy? In the simulation, the effect of the spot size could be estimated by a simple weighted sum of all dipole emitters, where the weights correspond to the intensity distribution of the illuminating spot. This would explain the large gap between simulation and experiment and prove that this is the major reason for the large gap between simulation and experiment.
2. The PL mapping in the top-right inset of Fig.4d exhibits radial symmetry, which is in contrast to the simulation in Fig. 4e. We wonder where this difference stems from? What polarization has been used in these measurements? Since the simulation uses linearly polarized light, non-polarized light in the experiment would explain these differences. However, if

this is the case, this should be clearly explained in the manuscript.
3. How sensitive is the directionality of the emission to vertical or lateral displacements of the emitters? As the Mie void mode is used for the emission, I would expect the displacements to have a small effect. This could be tested in a simulation and would improve clarity and increase the impact of the work.

Minor issues:
4. In Fig. 5a and/or b, the area covered by the monolayer should be indicated by an additional dashed boundary. It is only barely visible.
5. In Fig. 4, the comparison between experiment (d) and simulation (e) should be made more obvious in the figure title or y-axis labels. The same applies to Figs 5d,e, comparing void arrays and flat WSe2.
6. Scale bars or visible axes and ticks in Fig. 2a,b would improve clarity.

Reviewer #2

(Remarks to the Author)
I co-reviewed this manuscript with one of the reviewers who provided the listed reports. This is part of the Nature Communications initiative to facilitate training in peer review and to provide appropriate recognition for Early Career Researchers who co-review manuscripts.

Reviewer #3

(Remarks to the Author)
The manuscript reports enhancement and control of photoluminescence (PL) from WS2 coupled to Mie “void” resonators patterned in WSe2. Detailed electromagnetic simulations predict up to ~1700-fold PL enhancement relative to unpatterned (flat) WSe2. Experimentally, the authors fabricate the coupled structure and demonstrate ~600-fold PL enhancement along with out-of-plane directional emission. I appreciate the careful fabrication work and the interesting perspective on achieving PL enhancement using dielectric resonators based on 2D materials. However, before considering this manuscript for publication, I have two major concerns, as outlined below.
1. I am not fully convinced that the proposed Mie void resonators can provide stronger electromagnetic field enhancement than more conventional dielectric resonators (e.g., nanospheres, nanodisks, or nanopillars) made of low-loss materials. In my view, when a cavity mode is formed predominantly in air, the physical size of the resonator necessarily becomes large, resulting in relatively weak electromagnetic confinement. By contrast, conventional high-index dielectric resonators exhibit maximum electromagnetic fields inside the resonator; nevertheless, electromagnetic boundary conditions at the interface with the surrounding low-index medium (air) ensure the presence of strong near fields at the surface, which can be readily accessed by external emitters.
To support the central claim regarding the advantage of the Mie void geometry, a direct and quantitative comparison is therefore required, ideally based on electromagnetic simulations performed under identical conditions (i.e., using the same emitter model, excitation and collection geometry, resonance wavelength, and normalization procedure). For instance, the authors should benchmark the Purcell factor and/or LDOS, as well as near-field intensity enhancement, of the void resonator against representative high-index dielectric resonator geometries that are widely employed for photoluminescence enhancement.

2. In addition, the reported enhancement factors are referenced to flat WSe2, which may not be an appropriate baseline because emission can be significantly quenched on certain substrates or in close proximity to absorbing layers. Therefore, the magnitude of the enhancement may reflect suppression in the reference configuration rather than exceptional enhancement in the resonator configuration. A more rigorous assessment would compare against additional references on an optically benign substrate such as SiO2, and/or explicitly separate excitation enhancement, radiative-rate enhancement, and changes in quantum yield/collection efficiency.

Finally, while I acknowledge the technical challenge and value of covering a hole structure with 2D materials, related concepts have been reported previously by the same group. Given the high threshold for Nature Communications, and without clear evidence (particularly benchmarking simulations) that the PL enhancement and/or directionality are superior to conventional dielectric-resonator platforms, I cannot recommend publication in its current form.

Reviewer #4

(Remarks to the Author)
This manuscript describes a van der Waals nanophotonic platform coupling WS_2 monolayer excitons to WSe_2-based Mie voids, with a two-step fabrication method enabling independent void dimension control, a 600-fold WS_2 PL enhancement and a 2.6 dB forward-to-off-axis emission enhancement. While the experimental and simulation work is well-executed in adapting Mie voids to 2D van der Waals heterostructures, I do not recommend acceptance for publication, as key limitations prevent it from meeting the standards of a high-impact journal.

1.Mie resonance-based light emission control is a well-established research area, and the work merely adapts established dielectric Mie resonator frameworks to van der Waals heterostructures—with no novel mechanistic insights into light-matter interactions or distinct practical applications proposed. Its core design and operating principles closely build on prior research, with no impactful incremental advances to stand out in the field.

2.Reported performance also falls short of the current state-of-the-art. Many existing Mie resonance studies have demonstrated over 1000-fold PL enhancement and tunable forward/backward directional emission, whereas this work only achieves 600-fold enhancement and modest out-of-plane beaming, with no exploration of tunable radiation patterns, limiting its performance contribution to the field.
3.The selection of WSe_2 as the Mie void host is not clearly justified, and critical control experiments are missing. No rationale is provided for choosing WSe_2 over classic Mie materials like silicon, nor are comparative experiments conducted with these standard substrates. Additionally, the WS_2 monolayer on suspended WSe_2 voids may experience strain effect that modulates PL properties; without control experiments to rule this out, the observed enhancement cannot be definitively attributed to Mie resonance alone.
4.Foundational electromagnetic mode analysis for Mie voids is also absent. Characterizing electric/magnetic multipole modes and the Kerker effect—the physical basis of directional emission in such systems—is essential to understanding the optical effects observed, especially for the unique complementary geometry of Mie voids. This work only presents phenomenological observations, with no in-depth mechanistic analysis to support its core conclusions.
5.Finally, experimental results lack sufficient statistical generalizability. Mechanically exfoliated WS_2 monolayers have inherent structural inhomogeneities (stress variations, defects, interfacial impurities) that significantly impact PL properties, yet the study only presents results from limited samples and measurement positions, with no systematic statistical analysis across multiple voids or samples. This limited sampling may introduce bias from random inhomogeneities, reducing the reliability and generalizability of the study’s conclusions.

Version 2:

Reviewer comments:

Reviewer #1

(Remarks to the Author)
When I agreed to review the rebuttal version of this manuscript, I expected that the revised submission would contain substantial modifications addressing the concerns raised during the initial review process. In particular, given the significant criticisms expressed by Reviewers 2 and 3 regarding the impact and broader perspective of the work, I anticipated additional experimental evidence or a clearer demonstration of the manuscript’s significance. However, while the text has been revised in several places, the main manuscript itself has changed only marginally, with most additions appearing in the Supporting Information.

I have carefully considered both the authors’ responses and the revised manuscript. The responses are generally thoughtful and reasonable; however, my main concern is that these clarifications have not translated into corresponding improvements in the central message or impact of the manuscript itself. The extent of the rebuttal document, 19 pages in total, reflects the substantial nature of the questions raised during review. Nevertheless, the revisions do not, in my opinion, sufficiently address the key concerns regarding significance and positioning of the work.

After reconsidering the manuscript in light of the first-round reports and the rebuttal, I arrive at a conclusion similar to that of Reviewers 2 and 3. The experimental results are interesting and technically sound, but I remain unconvinced that the work demonstrates the level of conceptual advance or broad impact expected for publication in Nature Communications. In particular, the manuscript does not clearly establish what distinguishes this approach from existing concepts or where it offers a decisive advantage over previously demonstrated systems. Since stronger enhancement factors have already been reported using solid-particle-based approaches, I believe the manuscript would benefit from a clearer experimental demonstration of the unique opportunities enabled by the present concept which is clearly missing.

I understand the authors’ argument regarding the simultaneous enhancement of excitation and emission processes. However, given that the demonstrated performance appears less efficient than several existing approaches in the literature, I view this more as an important specialized contribution than as a broadly transformative advance.

Since the first review round, I have also become aware of two recently published papers that report closely related experiments and which, to the best of my knowledge, are not cited in the present manuscript: Light–matter interaction in van der Waals heterostructures with Mie voids (Advanced Photonics 8, 026002 (2026)) and Mie-Void-Enabled WS_2 Excitonic Emitters with Nanoscale Footprints (Nano Letters 26, 1888 (2026)). These publications further reduce the perceived novelty and impact of the present work. At the same time, I recognize that these studies may have been conducted on similar timelines, and therefore questions of priority and novelty are ultimately editorial considerations.

Comparing these recent publications with the current manuscript also influenced my assessment of the work’s scope and contribution. The Advanced Photonics paper investigates both photoluminescence enhancement and second-harmonic generation while employing resonance tuning through systematic depth variation, thereby avoiding the rather unconventional normalization procedure used in the present manuscript. This comparison also highlights that the newly introduced fabrication method for depth control shown in the current mansucript, while technically interesting, appears somewhat peripheral to the manuscript’s main focus on photoluminescence enhancement. Ultimately, the manuscript centers on two photoluminescence-related experiments, one addressing enhancement and one radiation control, which, although interesting, do not in my view reach the threshold of general significance required for publication in Nature Communications.

Regarding the revised version itself, I have two additional comments:
1. I am puzzled by the simulation results involving the Si disk. There is substantial literature precedent demonstrating enhanced emission in related systems, and it is therefore unclear why the presented simulations appear to indicate the opposite behavior.
2. I do not fully understand the significance of the phasor representation introduced in Fig. 5h, which appears to be the only substantial addition to the main text. Its role and physical interpretation are only minimally explained, making it difficult to assess its relevance to the manuscript's central conclusions.

Taking the manuscript, the rebuttal, and the broader literature context into account, I cannot recommend publication in Nature Communications. My concerns are not related to the validity of the experimental results, but rather to the overall level of conceptual advance and impact. In my opinion, the work would be more appropriately suited for a specialized journal, and I could envision a transfer to a venue such as Communications Physics.

Reviewer #2

(Remarks to the Author)
I co-reviewed this manuscript with one of the reviewers who provided the listed reports. This is part of the Nature Communications initiative to facilitate training in peer review and to provide appropriate recognition for Early Career Researchers who co-review manuscripts.

Reviewer #3

(Remarks to the Author)
The authors have addressed the major concerns raised by the reviewers by providing additional calculation results. I believe that the manuscript is now suitable for publication in Nature Communications.

Open Access This Peer Review File is licensed under a Creative Commons Attribution 4.0 International License, which permits use, sharing, adaptation, distribution and reproduction in any medium or format, as long as you give appropriate credit to the original author(s) and the source, provide a link to the Creative Commons license, and indicate if changes were made.
In cases where reviewers are anonymous, credit should be given to 'Anonymous Referee' and the source.
The images or other third party material in this Peer Review File are included in the article's Creative Commons license, unless indicated otherwise in a credit line to the material. If material is not included in the article's Creative Commons license and your intended use is not permitted by statutory regulation or exceeds the permitted use, you will need to obtain permission directly from the copyright holder.
To view a copy of this license, visit https://creativecommons.org/licenses/by/4.0/

We sincerely thank all four reviewers for their thorough evaluation of our manuscript and for their many insightful and constructive comments. We have carefully considered every point and provide below a detailed, point-by-point response to all comments. To address the concerns raised, we have substantially revised the manuscript, including clarifications of the conceptual advance and physical interpretation, improvements to the discussion and figure presentation, and the addition of new simulations and experimental analyses. Our responses are shown in green, whereas all revisions made in the main manuscript and SI are shown in red.

Reviewer #1-2:

The authors present a nanophotonic platform that utilizes Mie voids to solve a persistent challenge in dielectric nanoantennas: the fact that maximum electromagnetic fields are typically confined into high-index materials rather than accessible at the surface. By inverting the Mie geometry, the authors demonstrate significant photoluminescence (PL) enhancement and directional emission.

The work is innovative and well-written. Overall, the experiments and the presentation are of high quality, and I feel that, in principle, publication would be possible in Nature Communications. However, I have two major points, which I feel need clarification before publication can be considered. Moreover, there are a few important but minor additional questions/comments.

We thank the reviewers for the overall positive assessment of our work. Below we address the points raised.

Comment 1: The PL enhancement is very impressive; however, the observed values do surprise me. Intuitively, I would have expected significantly smaller values, also when comparing to published work on coupling emitters to plasmonic or dielectric systems. Just as a back-of-the-envelope calculation, I would look at the Purcell enhancement factor, which is proportional to lambda^3, Q, and the mode volume, given that the mode volume can be approximated with lambda^3 as well, the enhancement should linearly scale with the quality factor. For the excitation enhancement, I would also roughly assume a factor of Q, as this gives the enhanced fields compared to the far field. Overall, this would be proportional to Q^2, which gives (by looking at Fig 4e, Q approx. 4) a total of about 20. Surely this is just a very rough estimation, but it should give a feeling at least. The reported values are around 600, which is more than an order of magnitude larger and thus difficult for me to understand. I feel the authors need to make an intuitive case why the enhancement is so much larger as this really collides with my intuition and, as mentioned, other reported values in similar systems.

Response to Comment 1: We thank the reviewer for this thoughtful comment and agree that a simple estimate based on a moderate resonator Q would intuitively suggest a smaller enhancement. Indeed, when we normalize the excitation and emission efficiencies to the case of a low-index SiO_2 substrate, the simulated PL enhancement is approximately $60\times$ (Supplementary Section 1, Fig. S1), which is consistent with the order-of-magnitude estimate provided by the reviewer. In the main text, however, the enhancement is defined relative to a flat unstructured WSe_2 region [Eq. (1)], where the excitation and emission processes are

suppressed due to the high-index and absorptive nature of the substrate. This quenched reference leads to a larger enhancement, reaching up to ~ 600 × in experiment. We emphasize that this normalization is chosen because it provides a consistent experimental baseline within the same sample and reflects the relevant substrate reference for void geometries, which rely on high-index materials to confine electromagnetic fields.

To clarify this point, we now include the SiO_2-referenced comparison in a new Supplementary Section 1, Fig. S1 and have added the corresponding explanation in the main text:

Figure 2c shows that a void with a depth of 180 nm and a diameter of 520 nm yields the highest enhancement, reaching a 1700-fold increase in PL compared to unpatterned flat WSe_2 and around 60-fold increase compared to flat SiO_2 substrate (Supplementary Section 1, Fig. S1). The larger enhancement relative to WSe_2 arises because the high-index and absorptive substrate suppresses both the excitation and emission processes, leading to a quenched reference signal. We use flat WSe_2 as the reference, as it provides a consistent experimental baseline within the same sample and reflects the high-index material platform required for realizing void resonators.

The new Supplementary Section 1 and Fig. S1 are given below:

SI 1. Enhancement for SiO_2 substrate

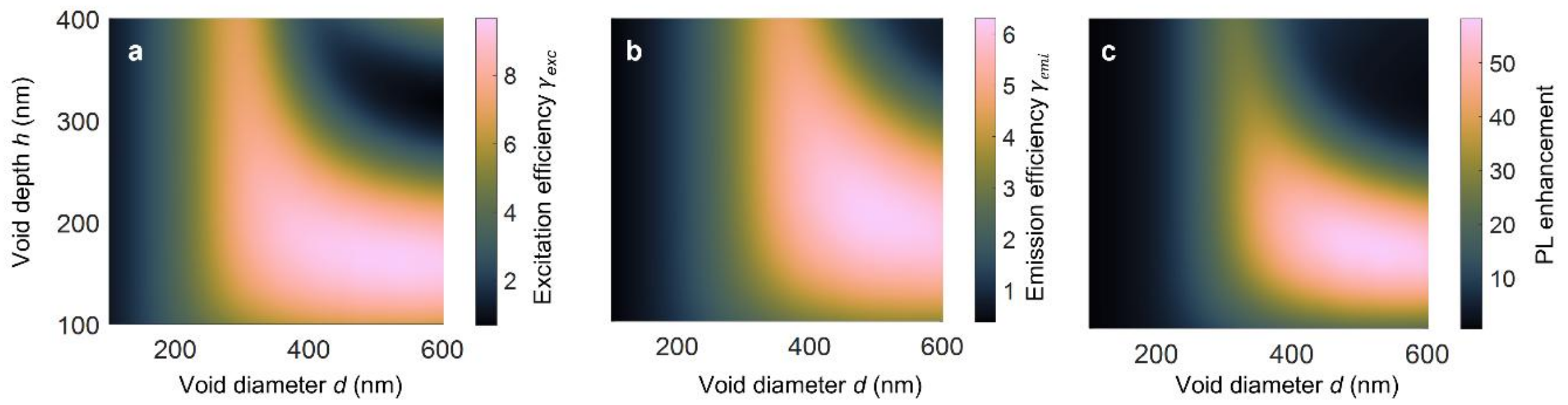

Fig. S1: Simulated enhancements normalized to SiO_2 substrate. a, Excitation efficiency γ_{exc}, b, emission efficiency γ_{emi} and c, resulting PL enhancement as a function of void diameter d and depth h for the dipole-on-void system normalized by dipole on flat semi-infinite SiO_2. The void resonators are still fabricated in bulk WSe_2.

The excitation efficiency, emission efficiency, and the resulting PL enhancement for a dipole placed on WSe_2 Mie voids are normalized to the case of flat SiO_2 substrate in Fig. S1. We observe that the PL enhancement reaches about 60×, which is lower than the 1700× enhancement observed when normalizing with flat WSe_2 (shown in Fig. 2 of the main text). The increased PL enhancement relative to flat WSe_2 originates from the quenching nature of the high-index and absorptive properties of WSe_2, which suppresses the excitation and emission processes.

Comment 2: One of the key points of the manuscript is the manipulation of the emission properties, which is a physically interesting effect. Again, intuitively, it is evident that the emission pattern should be modified due to the coupling to the void resonance. Measuring back-focal-plane images is a very smart idea for such experiments, however, due to the nature of the experiment, I feel some ambiguities need to be addressed. Most importantly, a model

and/or simulation of the radiation pattern is needed. Only such a model, predicting the emission pattern, can convincingly rule out other possible explanations as well as the influences of the experimental challenges, mainly the “normalization” due to the fact that most voids are not covered with the WS2 monolayer. Also, I wonder if there might be phased-array-like effects, which would also modify the emission pattern? Also, I wonder, what limits the sport size? Should a smaller aperture in the beam path not allow a smaller interrogated region?

Response to Comment 2: We thank the reviewer for this comment and agree that a quantitative model of the radiation pattern would be helpful to substantiate the experimental observations. We have therefore developed a numerical model of the emission process and included new simulation results in the revised manuscript (see new Fig. 5g–h). Specifically, we perform full-wave electromagnetic simulations in which the emitter is modeled as an electric dipole positioned at the opening of the void. From these simulations, we extract the far-field radiation pattern by evaluating the emitted power on a hemispherical surface. The resulting angular emission profile shows forward-directed beaming, in agreement with the experimentally measured back-focal-plane images (Fig. 5f). This provides evidence that the observed directionality arises from the coupling between the emitter and the void resonance.

To further clarify the physical mechanism, we perform a Cartesian multipole decomposition of the induced (scattered) field (see new Methods section). This analysis shows that the emission can be accurately described by a small set of electric and magnetic dipole and quadrupole contributions. Importantly, the decomposition enables a direct interpretation of the directionality in terms of interference between the electric dipole emitter and the induced multipoles of the void. As visualized in the newly added phasor diagram (Fig. 5h), the forward emission results from constructive interference between multiple multipolar contributions, while the induced electric dipole partially compensates the background field. This provides a clear physical picture of the emission shaping mechanism.

Regarding the possibility of phased-array-like effects, we note that both the simulations and the multipole analysis are performed for a single emitter–void system and already reproduce the observed directional emission. This indicates that the effect is primarily governed by local emitter–resonator coupling rather than collective array interactions.

Finally, regarding the spot size, we use Köhler illumination to uniformly probe the region of the void array that is homogeneously covered by the monolayer, ensuring that the measured angular signal originates exclusively from the monolayer-on-void configuration across the interrogated area. After collection, an aperture is placed at a plane conjugate to the sample to improve spatial selectivity and enhance the signal-to-noise ratio. As noted by the reviewer, in principle a smaller aperture should further improve spatial selectivity. However, in practice we observe that reducing the iris diameter below a certain threshold introduces fringe-like features in the Fourier (back-focal-plane) image. We attribute these fringes to diffraction effects arising from the finite size of the aperture. In addition, further closing the iris significantly reduces the collected signal, to the point where the signal-to-noise ratio of the back-focal-plane measurements cannot be recovered by increasing the exposure time. We therefore chose an aperture diameter that provides a practical compromise: small enough to remain within a

uniformly monolayer-covered region of the array, yet large enough to avoid diffraction artifacts and to maintain an adequate signal-to-noise ratio.

To clarify this point, we now provide a new Figure 5 with a revised figure caption:

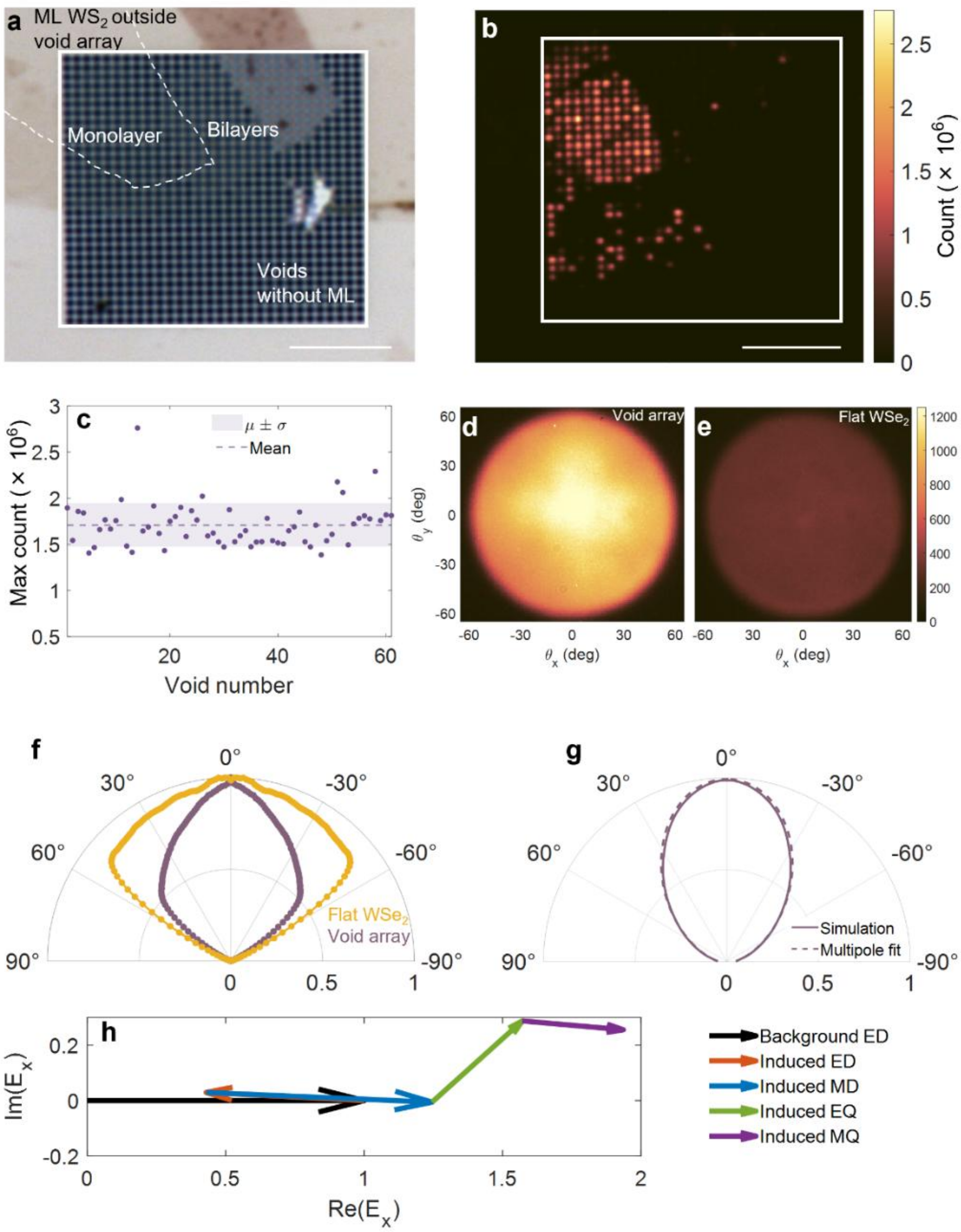

Fig. 5. Void-enabled directional emission. **a**, Optical image of a WS_2 monolayer (white dashed line) on a void array. Scale bar: 5 μm. **b**, PL map of the same region, showing brighter emission from monolayer-on-void regions. Scale bar: 5 μm. **c**, Maximum PL count of individual voids covered by monolayer WS_2. The black dashed line indicates the sample mean (μ) of the maximum PL intensity, and the shaded band shows the standard deviation (±σ) around the mean. **d**, Back-focal-plane image of a monolayer-on-array configuration. **e**, Back-focal-plane image of a monolayer-on-flat WSe_2 configuration. **f**, Azimuthally averaged one-dimensional angular PL profiles (normalized), comparing the void-array emission with the flat WSe_2 reference. **g**, Simulated (solid) and multipole-reconstructed (dashed) far-field emission pattern for a void of dimensions h = 330 nm and d =400 nm. **h**, Phasor representation of the total electric far-field in the forward emission direction decomposed into different multipolar contributions.

We have also added the following explanation in the main text:

To gain further insight into the physical origin of this directional emission, we simulate the far-field radiation pattern of a dipole-excited isolated void with dimensions h = 330 nm and d = 400 nm, matching the experimental void dimensions, and perform a Cartesian multipole decomposition of the induced far-field (see Methods). As shown in Fig. 5g, the multipole-expanded field faithfully reproduces the simulated angular distribution, confirming that the radiation can be captured by a finite set of electric and magnetic dipole and quadrupole contributions. The agreement between simulation and reconstruction demonstrates that the forward-directed emission arises from the coherent superposition of void-supported multipoles, which are excited by the electric dipole.

The interference mechanism underlying this directionality can be elucidated through a phasor representation of the electric field evaluated along the forward direction (Fig. 5h). The field contributions from the background electric dipole (ED) and the induced multipoles are represented as complex vectors and summed sequentially. Notably, the induced ED partially opposes the background ED along the forward direction, while the induced magnetic dipole (MD), the induced electric quadrupole (EQ) and the induced magnetic quadrupole (MQ) contribute constructively. The resulting phasor sum yields an enhanced total field in the forward direction, illustrating that the observed beaming arises from the coherent interference between multiple multipolar contributions. This analysis provides a clear physical picture of how the void structure reshapes the emission pattern through multipole coupling.

We have also added a new Methods section that explains the simulation and multipole decomposition process:

The electromagnetic response of an isolated cylindrical void is computed using finite-element simulations in COMSOL Multiphysics in the frequency domain at a wavelength of λ = 610 nm. The system is excited by an $\hat{x}$-oriented electric dipole point source positioned laterally at the center and vertically at the opening of the void. The surrounding medium is air, and the computational domain is terminated by perfectly matched layers. The cylindrical void in WSe_2 is modeled using an impedance boundary condition. To perform the multipole decomposition, the electric field is sampled on a hemispherical surface in the air region of radius R = 2 µm centered at the dipole position. Simulations are performed both with and without the void structure, allowing extraction of the induced field as the difference between the total and background fields. Only the tangential components of the electric field are retained in order to isolate the far-field contribution.

The induced electric far-field is decomposed into Cartesian multipoles by fitting it to a linear combination of electric dipole, magnetic dipole, electric quadrupole, and magnetic quadrupole radiation patterns. The angular dependence of the induced field is modeled as [48]

$$E(\hat{n}) \propto \hat{n} \times (p \times \hat{n}) + \frac{1}{c}(m \times \hat{n})$$

$$+ \frac{ik}{c}\hat{n} \times (\hat{n} \times (Q^e \cdot \hat{n})) + \frac{ik}{6c}\hat{n} \times (Q^m \cdot \hat{n}),$$

where $\hat{n}$ denotes the observation direction, p and m are the electric and magnetic dipole moments, and Q^{e} and Q^{m} are symmetric, traceless quadrupole tensors. The multipole coefficients are obtained by solving a complex least-squares problem that minimizes the deviation between the simulated tangential field and the multipole expansion evaluated at all sampled directions. The quadrupole tensors are expanded in a basis of symmetric, traceless matrices, reducing the problem to a linear fit with a finite number of unknown coefficients.

Comment 3: The authors state that roughly 50% loss in predicted enhancement stems from the limited spot size of the diffraction-limited illumination laser. To what extent might other effects contribute to this discrepancy? In the simulation, the effect of the spot size could be estimated by a simple weighted sum of all dipole emitters, where the weights correspond to the intensity distribution of the illuminating spot. This would explain the large gap between simulation and experiment and prove that this is the major reason for the large gap between simulation and experiment.

Response to Comment 3: We thank the reviewer for this helpful suggestion. We agree that the influence of the diffraction-limited excitation spot could be evaluated explicitly. Accordingly, we have now added this analysis in the revised manuscript. We convolve the simulated spatial PL profile with a Gaussian kernel representing the measured excitation spot size, which reduces the peak enhancement from $\sim 1300\times$ to $\sim 730\times$, bringing it much closer to the experimental value of $\sim 600\times$. The procedure is presented in a new Supplementary Section 9, Fig. S9, where the excitation spot is modeled with a normalized Gaussian kernel of FWHM 0.59 μm. This analysis confirms that finite-spot spatial averaging is the major origin of the discrepancy between the idealized simulation and experiment.

We have added the following explanation in the main text:

To account for this finite spot size, we convolved the simulated spatial PL profile with a Gaussian kernel corresponding to the experimental illumination spot size, which reduces the peak enhancement at the void center to approximately $730\times$ (see Supplementary Section 9, Fig. S9).

The new Supplementary Section 9 and Fig. S9 are given below:

SI 9. Finite-spot-size averaging of the simulated spatial PL profile by Gaussian convolution

To account for the finite spatial extent of the excitation spot in the experiment, we convolved the simulated spatial PL profile with a Gaussian kernel corresponding to the measured spot size. For an illumination spot diameter of 1 μm, we approximate the spatial profile by a Gaussian with a full width at half maximum (FWHM) of 0.59 μm, corresponding to a standard deviation

$$\sigma = \frac{\mathrm{FWHM}}{2\sqrt{2\ln 2}} \approx \frac{0.59\ \mu\mathrm{m}}{2.355} \approx 0.25\ \mu\mathrm{m}.$$

The normalized Gaussian kernel is therefore written as

$$G(x) = \exp\left(-\frac{x^2}{2\sigma^2}\right), \qquad \tilde{G}(x) = \frac{G(x)}{\int G(x)\,dx},$$

and the finite-spot-averaged PL profile is obtained by convolution of the simulated point-dipole PL response $\mathrm{PL_{sim}}(x)$ with the Gaussian kernel,

$$\mathrm{PL_{conv}}(x_0) = \int \mathrm{PL_{sim}}(x)\ \tilde{G}(x - x_0)\,dx.$$

Figure S9a shows the normalized Gaussian kernel used for the averaging procedure, while Fig. S9b compares the original simulated PL profile with the Gaussian-convolved result. The point-dipole simulation predicts a maximum PL enhancement of about 1300× at the void center, whereas the finite-spot averaging reduces the enhancement to approximately 730 ×, much closer to the experimental value. This analysis shows that the experimentally relevant PL enhancement is significantly lower than the ideal point-dipole value because the illumination spot excites not only the void center but also the surrounding region, thereby spatially averaging the response.

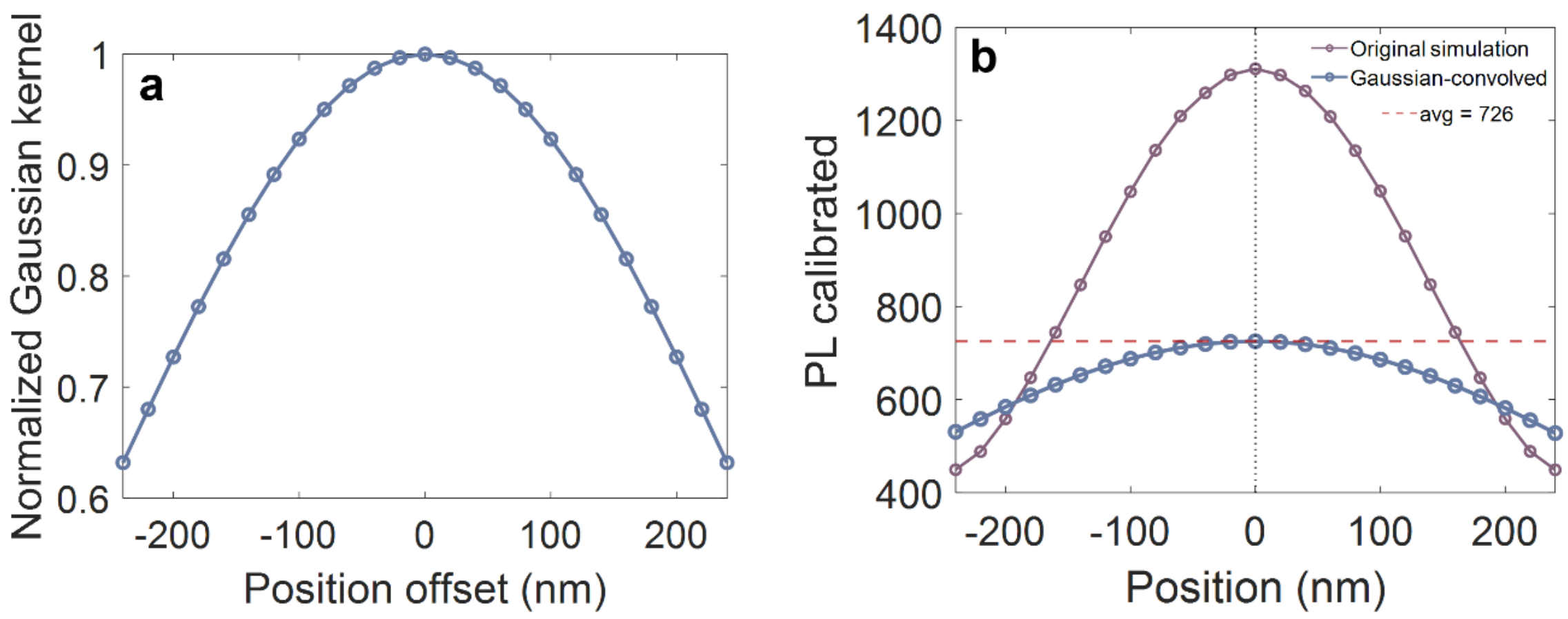

Fig. S9: **Finite-spot averaging of the simulated PL profile by Gaussian convolution. a,** Normalized Gaussian kernel used to model the spatial profile of the excitation spot, plotted as a function of position offset from the void center. **b,** Simulated calibrated PL profile across the void before and after convolution with the Gaussian kernel. The original point-dipole simulation exhibits a peak enhancement of about 1300× at the void center, whereas the Gaussian-convolved profile is broadened and reduced in amplitude, yielding a maximum enhancement of approximately 730 × at the center of the void resonator. The vertical dotted line marks the void center.

Comment 4: The PL mapping in the top-right inset of Fig. 4d exhibits radial symmetry, which is in contrast to the simulation in Fig. 4e. We wonder where this difference stems from? What polarization has been used in these measurements? Since the simulation uses linearly polarized light, non-polarized light in the experiment would explain these differences. However, if this is the case, this should be clearly explained in the manuscript.

Response to Comment 4: We thank the reviewer for this comment. As the reviewer rightly stated, the apparent discrepancy between the radial symmetry of the experimental PL map in Fig. 4d and the anisotropy of the simulated field profile in Fig. 4e stems from the excitation conditions. In the simulations, we use an x-polarized incident plane wave and an x-oriented

emitting dipole, which produces an asymmetry between the x- and y-directions. In the experiment, however, the PL map reflects an effective polarization-averaged excitation condition and therefore shows no preferred in-plane direction, resulting in a radially symmetric response.

We have now added this clarification to the manuscript:

The apparent asymmetry between the x- and y-directions in the simulated field profile arises from the use of x-polarized excitation in the calculations. In contrast, the experimental illumination is unpolarized, which results in a radially symmetric response in the mapping.

Comment 5: How sensitive is the directionality of the emission to vertical or lateral displacements of the emitters? As the Mie void mode is used for the emission, I would expect the displacements to have a small effect. This could be tested in a simulation and would improve clarity and increase the impact of the work.

Response to Comment 5: We thank the reviewer for this helpful suggestion. We address this point in Supplementary Section 6, Fig. S6 (Supplementary Section 2, Fig. S2 in the old manuscript). In line with the reviewer's comment, the simulations show that the emission efficiency γ_{emi} is only weakly affected by vertical displacement. When the dipole is kept laterally centered, γ_{emi} remains nearly constant for z-offsets of up to 20 nm above the void opening (Fig. S6a). The lateral displacement also causes weak change in emission efficiency for a displacement up to around 80 nm from the void center (Fig. S6c). Thus, the directional emission is relatively robust against vertical offsets, but more sensitive to in-plane misalignment, which mainly reduces the strength of the emitter–void coupling.

Comment 6: In Fig. 5a and/or b, the area covered by the monolayer should be indicated by an additional dashed boundary. It is only barely visible.

Response to Comment 6: We thank the reviewer for identifying the issue. A dashed boundary indicating the area covered by the monolayer is added to Fig. 5a and referred to in the figure caption. The revised Figure 5 is presented in our response to Reviewer 1, Comment 2.

Comment 7: In Fig. 4, the comparison between experiment (d) and simulation (e) should be made more obvious in the figure title or y-axis labels. The same applies to Figs 5d,e, comparing void arrays and flat WSe2.

We agree with the reviewer that these comparisons could be presented more clearly. We have revised the figure y-axis labels in Fig. 4, such that labels now read "PL enhancement (exp.)" and "PL enhancement (sim.)". In Fig. 5, the compared cases are now directly labeled as "Void array" and "Flat WSe_2". We believe these changes make the experiment–simulation and void–flat comparisons more immediate for the reader.

The revised Fig. 4 is provided below. The revised Figure 5 is presented in our response to Reviewer 1, Comment 2.

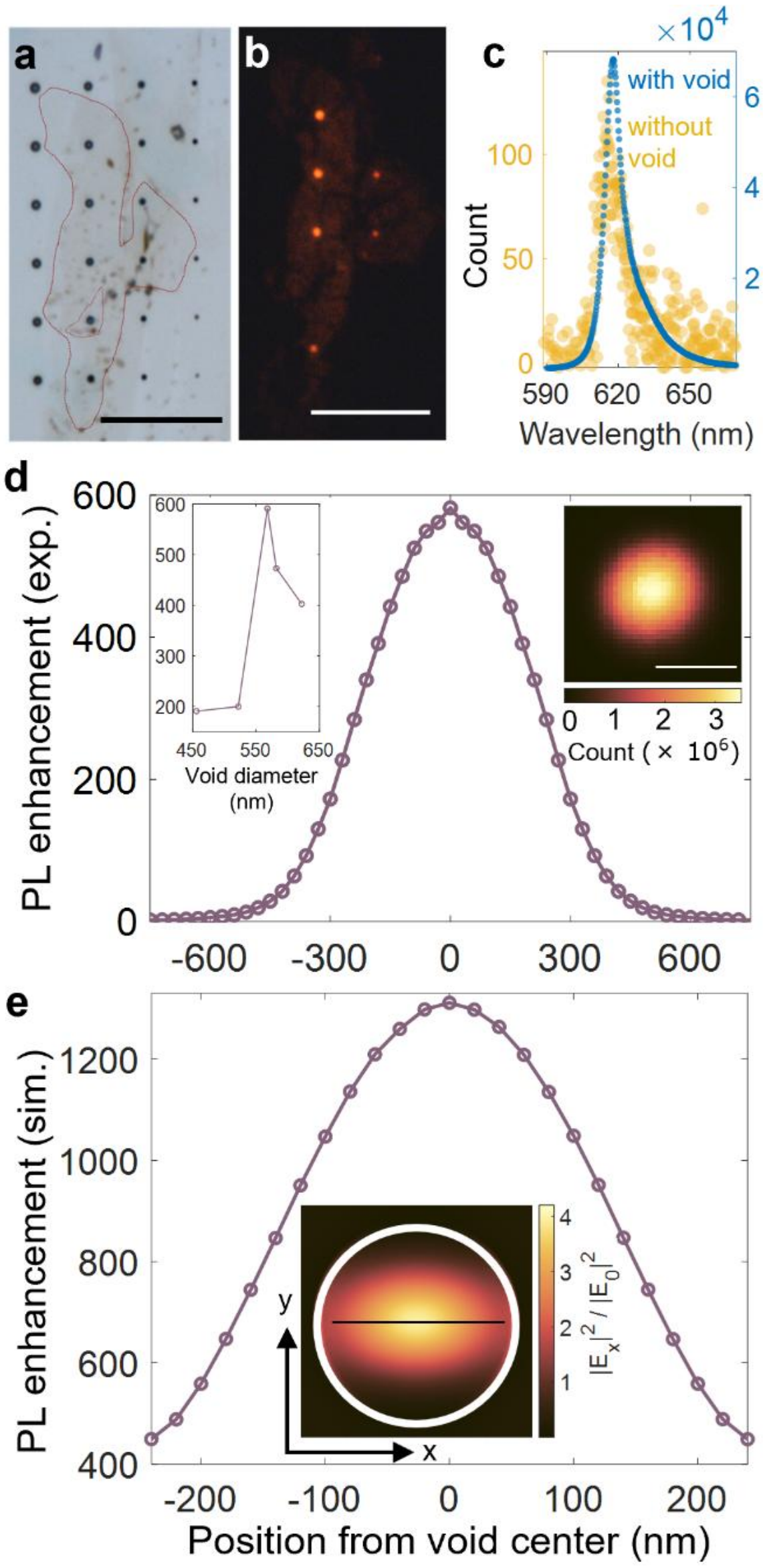

Comment 8: Scale bars or visible axes and ticks in Fig. 2a,b would improve clarity.

We thank the reviewer for this comment. Ticks are added on Fig. 2a,b. The revised Figure 2 is presented below.

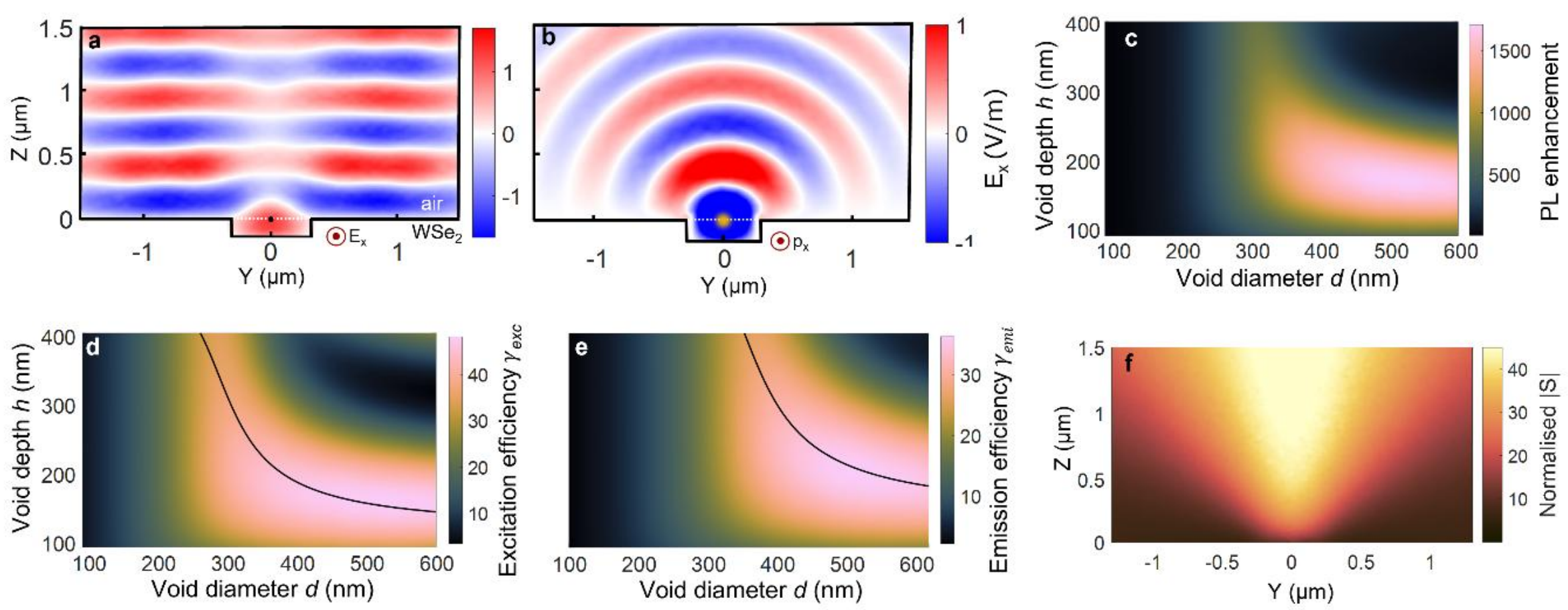

Reviewer #3:

The manuscript reports enhancement and control of photoluminescence (PL) from WS2 coupled to Mie "void" resonators patterned in WSe2. Detailed electromagnetic simulations predict up to ~1700-fold PL enhancement relative to unpatterned (flat) WSe2. Experimentally, the authors fabricate the coupled structure and demonstrate ~600-fold PL enhancement along with out-of-plane directional emission. I appreciate the careful fabrication work and the interesting perspective on achieving PL enhancement using dielectric resonators based on 2D materials. However, before considering this manuscript for publication, I have two major concerns, as outlined below.

We thank the reviewer for the careful assessment of our manuscript and for highlighting key aspects of the work. We address the reviewer's concerns in detail below.

Comment 1: I am not fully convinced that the proposed Mie void resonators can provide stronger electromagnetic field enhancement than more conventional dielectric resonators (e.g., nanospheres, nanodisks, or nanopillars) made of low-loss materials. In my view, when a cavity mode is formed predominantly in air, the physical size of the resonator necessarily becomes large, resulting in relatively weak electromagnetic confinement. By contrast, conventional high-index dielectric resonators exhibit maximum electromagnetic fields inside the resonator; nevertheless, electromagnetic boundary conditions at the interface with the surrounding low-index medium (air) ensure the presence of strong near fields at the surface, which can be readily accessed by external emitters. To support the central claim regarding the advantage of the Mie void geometry, a direct and quantitative comparison is therefore required, ideally based on electromagnetic simulations performed under identical conditions (i.e., using the same emitter model, excitation and collection geometry, resonance wavelength, and normalization procedure). For instance, the authors should benchmark the Purcell factor and/or LDOS, as well as near-field intensity enhancement, of the void resonator against representative high-index dielectric resonator geometries that are widely employed for photoluminescence enhancement.

Response to Comment 1: We thank the reviewer for this comment. To address the reviewer's concern, we now include a comparison with Si nanodisks in a new Supplementary Section 4, Fig. S4**,** using the same dipole-emitter model, excitation and emission wavelengths, collection geometry, and normalization procedure as in the void-resonator calculations. To enable the same normalization procedure, we place the Si nanodisk on the same substrate (WSe_2) as the void resonator.

The purpose of this comparison is not to claim that a void resonator universally outperforms every possible conventional dielectric geometry. Rather, our central point is that, for an atomically thin emitter coupled to an optical resonator, the cylindrical void geometry enables simultaneous optimization of excitation and emission processes, whereas in dielectric resonators these optima are mismatched. The new simulations support this point. For the Si nanodisk platform, the geometries that maximize the excitation and emission efficiencies do not coincide perfectly, so the PL enhancement is limited by incomplete overlap between the

two processes. As the emitter–resonator separation is increased to only a few nanometers, the excitation and emission optima shift further apart and the achievable enhancement drops. The Si nanodisk therefore becomes sensitive to small changes in resonator dimensions and gap size.

By contrast, in the void geometry the strongest field is exposed at the void opening, i.e. directly where the monolayer emitter resides, and the constructive-interference condition that maximizes the excitation field at the opening also governs efficient upward emission. As a result, the excitation and emission optima remain nearly aligned, making the PL enhancement both larger and more tolerant to fabrication variations. We now state this more explicitly in the main text and refer the reader to the benchmark in Supplementary Section 4.

We have now added this clarification to the manuscript:

For comparison, analogous simulations for Si nanodisks show that the excitation and emission efficiencies often peak at markedly different geometries, in many cases remaining far apart in parameter space, resulting in lower and geometry-sensitive PL enhancement (Supplementary Section 4, Fig. S4).

The Supplementary Section 4 and Fig. S4 are given below.

SI 4. Comparison with Si nanodisks

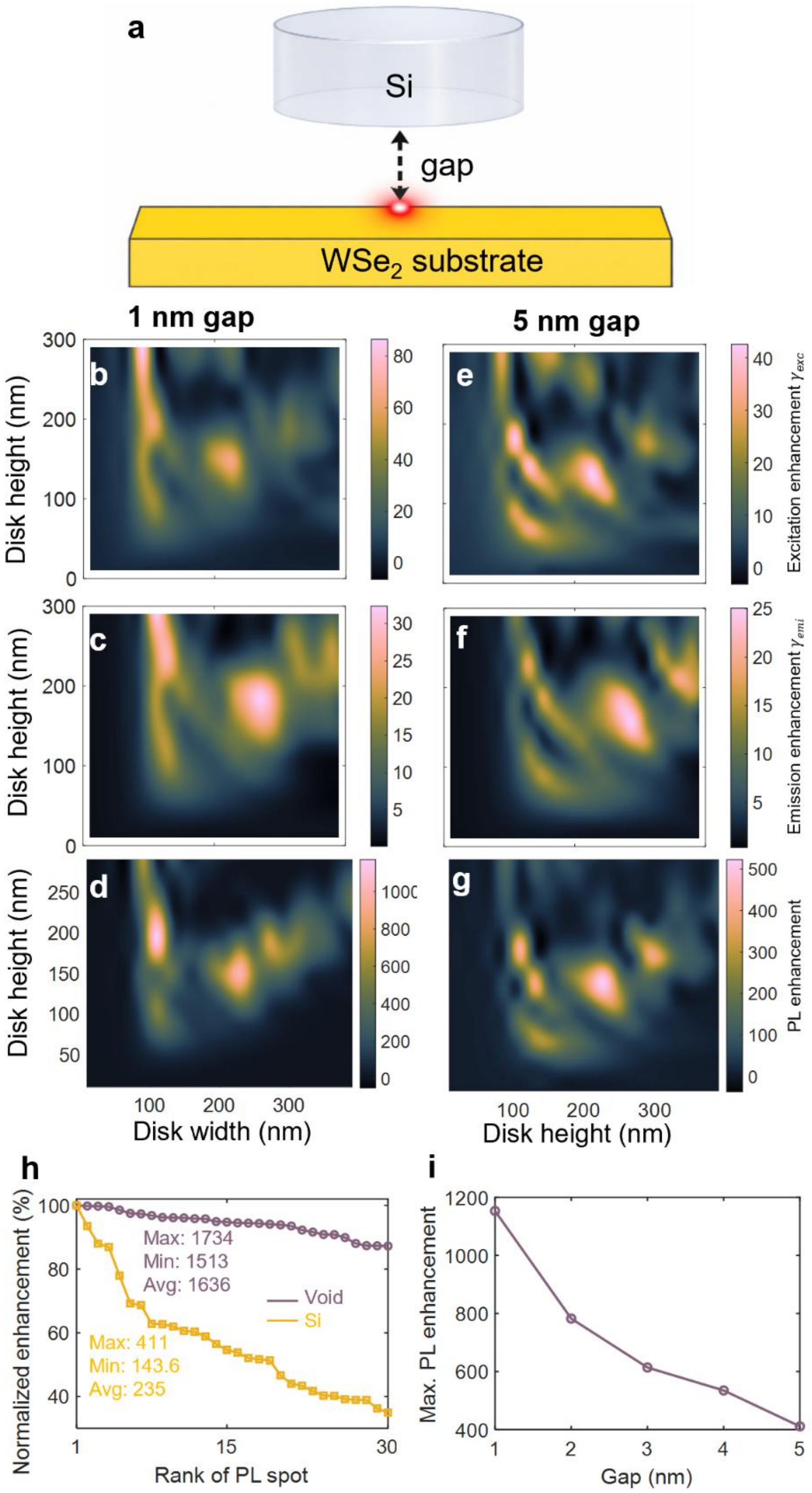

Fig. S4: Simulated enhancements for Si nanodisk separated from the emitter by a finite air gap. a, Schematic of the simulated geometry. **b–d,** Excitation enhancement, emission enhancement, and PL enhancement for a 1 nm gap. **e–g,** Same as in **b–d**, but for a 5 nm gap. **h**, Normalized PL enhancement of the top 30 brightest parameter combinations for the void resonator and the Si nanoresonator. **i**, Maximum PL enhancement of the Si nanoresonator as a function of emitter–resonator gap.

To assess the performance of a dielectric resonator, we simulate the excitation enhancement, emission enhancement, and resulting PL enhancement for a dipole placed on a WSe_2 substrate beneath a Si nanodisk. The dipole position is kept fixed at the WSe_2 surface, while the nanodisk height and diameter are varied to generate two-dimensional parameter maps. In addition, the vertical separation between the substrate and the Si nanodisk is varied to represent the finite spacer (like hBN) thickness between the monolayer emitter and the resonator (Fig. S4a). Figure S4b-d and Fig. S4e-g present two representative cases corresponding to emitter-resonator gaps of 1 and 5 nm, respectively.

Several clear trends emerge from these simulations. First, even for the 1 nm-gap case, the geometries that maximize the excitation enhancement and those that maximize the emission enhancement do not fully coincide (Fig. S4b,c). As a result, the condition for maximum PL enhancement is limited by an incomplete overlap between the excitation and emission optima (Fig. S4d). Second, when the gap is increased to 5 nm, this mismatch becomes more pronounced (Fig. S4e,f). The optimal regions for excitation and emission shift further apart in parameter space, and the resulting PL enhancement shows a clear reduction in the achievable maximum value (Fig. S4g). The Si nanodisk therefore exhibits strong sensitivity to resonator geometry, with only a narrow region of parameter space yielding high PL enhancement.

This sensitivity originates from the spatial distribution of the resonant field. Unlike voids, in the Si nanodisk platform, the strongest optical field is confined predominantly inside the resonator, whereas the emitter is located outside the resonator on the WSe_2 surface. Consequently, even a small increase in the emitter-resonator gap significantly reduces the spatial overlap between the emitter and the localized near field. This lowers the combined excitation and emission efficiencies, leading to a decrease in the PL enhancement.

To quantify the fabrication tolerance of the different resonator platforms, we rank the 30 brightest PL enhancement values obtained from the two-dimensional parameter sweeps in resonator height and diameter. Since both parameters were varied using a 20 nm step size, these top 30 values correspond to the 30 grid points in the (h, w) parameter space that yield the highest simulated PL enhancement. For each platform, the ranked PL enhancement values are normalized to the brightest value in that same dataset, i.e. $\mathrm{PL_{norm}}(n) = 100 \times \mathrm{PL}(n)/\mathrm{PL}(1)$. As shown in Fig. S4h, the void resonator exhibits a slower decay across the top 30 ranked values than the Si nanodisk. For the void resonator, the top 30 PL enhancement values lie between 1513 and 1734, with an average of 1636, indicating that high PL enhancement is maintained across a relatively broad region of geometry space. The slow decay demonstrates that many nearby geometries perform similarly well and therefore that the void geometry is more tolerant to fabrication-induced deviations. By contrast, for the Si nanodisk with a 5 nm gap, the top 30 values span from 144 to 411, with an average of 235, and the normalized curve decreases much more rapidly. This steep decay demonstrates a stronger sensitivity to dimensional variations for the Si nanodisk.

The gap dependence of the Si nanoresonator is summarized in Fig. S4i, where the maximum PL enhancement is plotted as a function of emitter-resonator gap from 1 to 5 nm. The maximum

PL enhancement decreases monotonically from 1153 at 1 nm gap to 411.1 at 5 nm gap. This trend shows that the Si nanodisk is highly sensitive to emitter-resonator separation.

Comment 2: In addition, the reported enhancement factors are referenced to flat WSe2, which may not be an appropriate baseline because emission can be significantly quenched on certain substrates or in close proximity to absorbing layers. Therefore, the magnitude of the enhancement may reflect suppression in the reference configuration rather than exceptional enhancement in the resonator configuration. A more rigorous assessment would compare against additional references on an optically benign substrate such as SiO2, and/or explicitly separate excitation enhancement, radiative-rate enhancement, and changes in quantum yield/collection efficiency.

Response to Comment 2: We thank the reviewer for this comment. We kindly refer the reviewer to our response to Reviewer 1, Comment 1, since it concerns the same point and explains the choice of reference for the reported enhancement factors.

Comment 3: Finally, while I acknowledge the technical challenge and value of covering a hole structure with 2D materials, related concepts have been reported previously by the same group. Given the high threshold for Nature Communications, and without clear evidence (particularly benchmarking simulations) that the PL enhancement and/or directionality are superior to conventional dielectric-resonator platforms, I cannot recommend publication in its current form.

Response to Comment 3: We thank the reviewer for this comment. We would like to clarify that, while the two studies share a similar material platform and fabrication methodology, the underlying scientific objectives are fundamentally different. In our previous work, the transferred layer was optically passive and used to modify the void geometry, whereas in the present study the monolayer acts as an active emitter whose photoluminescence is coupled to the void resonance.

More importantly, the revised manuscript now contains direct benchmarking and additional physical analysis addressing the reviewer's concerns. In particular, we have added: (i) a SiO_2-referenced comparison in Supplementary Section 1 to clarify the magnitude of the enhancement relative to a low-index substrate, and (ii) a direct comparison with Si nanodisks in Supplementary Section 4 under identical emitter and normalization conditions. In addition, the main manuscript now includes a multipolar analysis of the directional emission, linking the experimentally observed beaming to the underlying resonant response of the void geometry (Section: Directional emission using Mie voids, Fig. 5g,h). Taken together, these additions provide direct comparison and physical insight demonstrating that the observed enhancement and directionality originate from the void resonators, and establish their advantage in enabling simultaneous optimization of excitation and emission processes.

Reviewer #4

This manuscript describes a van der Waals nanophotonic platform coupling WS_2 monolayer excitons to WSe_2-based Mie voids, with a two-step fabrication method enabling independent void dimension control, a 600-fold WS_2 PL enhancement and a 2.6 dB forward-to-off-axis emission enhancement. While the experimental and simulation work is well-executed in adapting Mie voids to 2D van der Waals heterostructures, I do not recommend acceptance for publication, as key limitations prevent it from meeting the standards of a high-impact journal.

Comment 1: Mie resonance-based light emission control is a well-established research area, and the work merely adapts established dielectric Mie resonator frameworks to van der Waals heterostructures—with no novel mechanistic insights into light-matter interactions or distinct practical applications proposed. Its core design and operating principles closely build on prior research, with no impactful incremental advances to stand out in the field.

Response to Comment 1: We thank the reviewer for this comment. In the original manuscript, we already identified the physical mechanism underlying the enhanced and directional emission, namely that the same constructive-interference condition governing the local field enhancement at the emitter position also governs the forward-directed emission, enabling simultaneous optimization of excitation and emission processes.

In the revised manuscript, we have further clarified and strengthened both the mechanistic insight and the practical relevance of the platform. First, to provide a more explicit physical description of the directional emission, we now include a multipolar decomposition of the emitted field (Section: Directional emission using Mie voids, Fig. 5g,h), which identifies the beaming in terms of interference between specific multipolar contributions of the void resonance (see also response to Reviewer 1, Comment 2 for details).

Second, to assess the practicality of the void geometry, we have added benchmarking against conventional Si nanodisk resonators under identical excitation, emission, and normalization conditions (new Supplementary Section 4, Fig. S4). These results show that, in the experimentally relevant configuration of an emitter located outside the resonator, the void geometry enables simultaneous optimization of excitation and emission processes over a broader parameter range. This leads to higher achievable PL enhancement and improved tolerance to geometric variations compared to the dielectric resonator case (further detailed in our response to Reviewer 3, Comment 1).

Together, these additions demonstrate that the present work goes beyond adaptation of existing Mie resonator concepts by establishing a distinct mechanism for achieving simultaneous enhancement and directionality in emitter-coupled Mie voids, along with a quantitative comparison to conventional dielectric platforms.

Comment 2: Reported performance also falls short of the current state-of-the-art. Many existing Mie resonance studies have demonstrated over 1000-fold PL enhancement and tunable forward/backward directional emission, whereas this work only achieves 600-fold

enhancement and modest out-of-plane beaming, with no exploration of tunable radiation patterns, limiting its performance contribution to the field.

Response to Comment 2: We thank the reviewer for this comment. We would like to clarify that the central goal of this work is not to achieve the highest reported photoluminescence enhancement or to explore tunable radiation patterns, but rather to demonstrate that Mie void resonators can simultaneously enhance and direct emission from an atomically thin emitter located outside the resonator. In this context, the key result is that the void geometry enables simultaneous enhancement of excitation and emission processes, leading to strong photoluminescence enhancement together with directional emission in a single structure. This behavior arises from the constructive-interference condition identified in the manuscript and is further supported by the additional analysis introduced in the revised version.

To place the performance in context, we have also included benchmarking against conventional dielectric resonators (Supplementary Section 4, Fig. S4), which shows that the void geometry provides comparable enhancement while offering a broader parameter space for achieving combined excitation and emission efficiency.

We therefore believe that the significance of the present work lies in establishing and experimentally validating this mechanism in Mie void resonators, rather than in optimizing a single performance metric. In doing so, it provides both the physical foundation and the broader motivation for continued exploration of Mie void resonators as a versatile platform for manipulating two-dimensional excitons.

Comment 3: The selection of WSe_2 as the Mie void host is not clearly justified, and critical control experiments are missing. No rationale is provided for choosing WSe_2 over classic Mie materials like silicon, nor are comparative experiments conducted with these standard substrates. Additionally, the WS_2 monolayer on suspended WSe_2 voids may experience strain effect that modulates PL properties; without control experiments to rule this out, the observed enhancement cannot be definitively attributed to Mie resonance alone.

Response to Comment 3: We thank the reviewer for this important comment. We agree that the choice of WSe_2 as the Mie void host should be stated more explicitly, and that possible strain-induced PL changes must be ruled out more clearly.

We choose WSe_2 as the host material, because its high in-plane refractive index and non-negligible extinction coefficient in the visible lead to strong reflection at the void boundaries, enabling the formation of Mie void resonances. In addition, its layered nature enables deterministic exfoliation, which allows independent control over the void depth and diameter, as discussed in relation to Figure 3.

We have added the following line in the main manuscript:

We choose WSe_2 as the host material because its high refractive index and non-negligible extinction coefficient in the visible lead to strong reflection at the void boundaries, enabling the formation of Mie void resonances [31].

Regarding strain, we now address this point by adding additional control measurements to rule out strain as the dominant origin of the observed PL enhancement. In Supplementary Section 8 and Fig. S8, we compare three stacking configurations on the same void platform that result in different strain conditions. We find that the PL enhancement remains similar across all configurations, indicating that the enhancement is predominantly due to the coupling of excitons to the Mie void resonators.

Previous studies (cited in Supplementary Section 8) also suggest that the required strain to significantly impact the PL is substantially higher than the maximum observed strain in our devices. Moreover, the spectral measurements shown in Figure 4c do not indicate any prominent spectral shift of the monolayer-on-void case compared to the flat WSe_2, which is a signature for strain affected emissions. We therefore conclude that the dominant effect arises from the coupling of excitons to the Mie void resonators.

We have added the following line in the main text:

Control experiments show that the observed enhancement predominantly arises from coupling to the void resonances rather than strain-induced effects (Supplementary Section 8, Fig. S8).

The new Supplementary Section 8 and Fig. S8 are given below:

SI 8. Effect of local strain at the void-monolayer interface

We have performed control experiments to assess whether strain in WS_2 impacts the observed PL enhancement. Figure S8a shows a two-dimensional schematic of the three stacking configurations realized on the same void platform, where the gray region represents the void array. The three cases correspond to a monolayer supported by bottom hBN only, a monolayer covered by top hBN only, and a monolayer fully encapsulated between top and bottom hBN. Figure S8b shows the optical image of the void arrays with different dimensions and identifies the spatial regions corresponding to the different stacking configurations. Figure S8c presents the PL maps measured from these regions superimposed on the bright-field image of the void arrays. Figure S8d shows AFM line cuts comparing their topographies.

The AFM data indicate that the different stacking configurations produce different local strain conditions in the monolayer. For the fully encapsulated case and the bottom-hBN-supported case (case 1 and 3) we do not observe periodic strain modulation induced by the voids. In contrast, when only top hBN is present (case 2), a periodic strain modulation of up to 0.16% is observed, estimated using $\varepsilon \approx \frac{2}{3}\left(\frac{\delta}{r}\right)^2$, where r is the void radius and δ is the maximum deflection of the monolayer at the void center.

Such strain levels are relatively small, and prior studies indicate that significantly larger strain (>0.5%) is required to produce pronounced PL enhancement in TMDC monolayers [2-4]. To directly assess the impact on our system, we analyze a high-resolution PL map of the upper void array in Fig. S8e, where seven circular regions of identical area are selected across different parts of the array, spanning the different stacking configurations. For each region, the integrated PL intensity is plotted in Fig. S8f. The total PL intensity remains within a similar

range across all regions, with a mean value of 1.9×10^9 counts and a relative standard deviation of 9.7%.

These results show that variations in local strain do not lead to significant changes in the PL intensity, indicating that strain is not the dominant factor governing the observed enhancement. Instead, the enhancement is primarily determined by coupling of the excitons to the Mie void resonators.

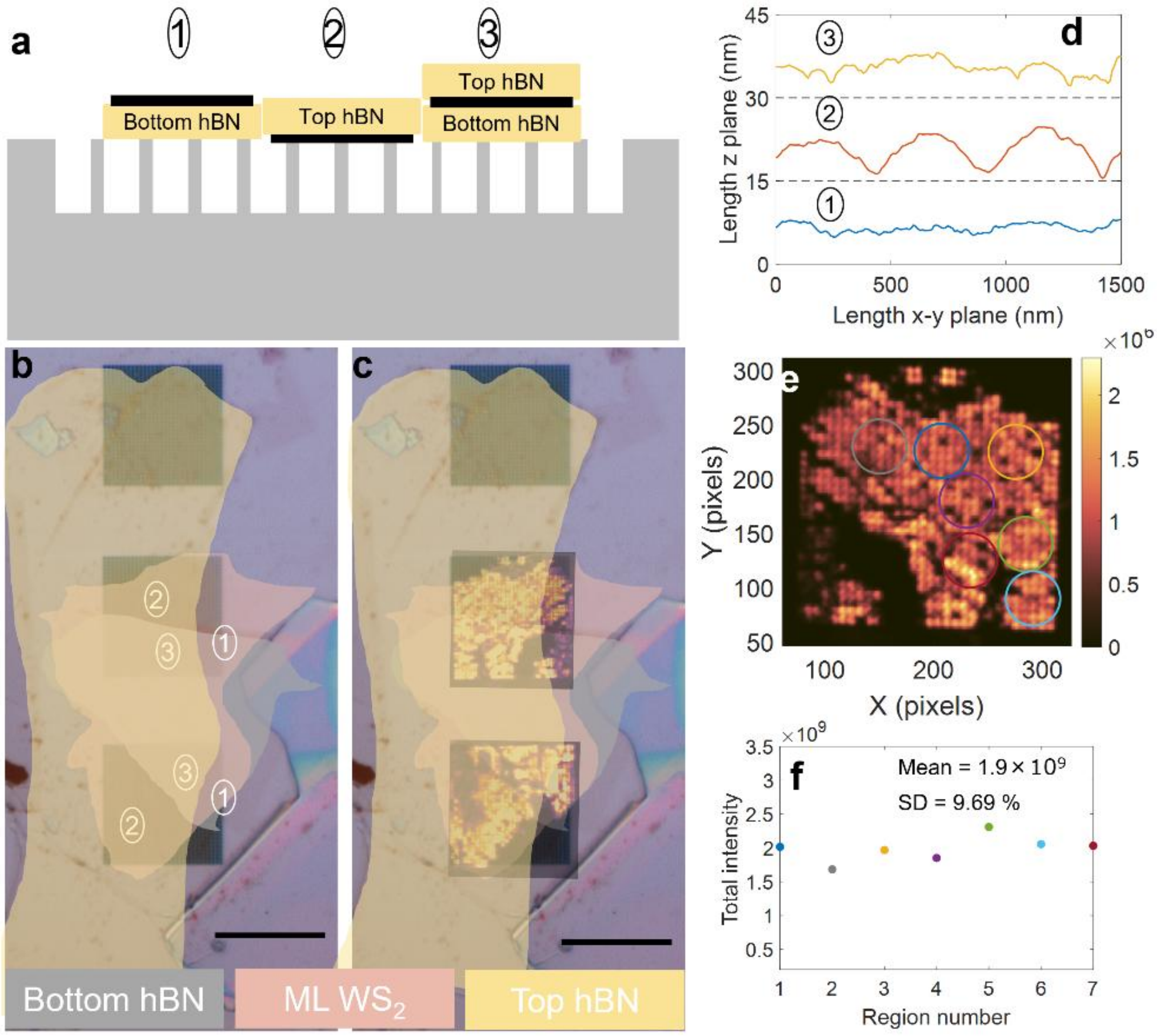

Fig. S8: Photoluminescence mapping comparing different hBN stacking configurations on the void array device. **a**, Two-dimensional schematic of three local stacking configurations on the same void platform, where the gray patterned region represents the void array: (1) monolayer on bottom hBN only, (2) monolayer with top hBN only, and (3) monolayer fully encapsulated by top and bottom hBN. **b**, Schematics on the optical image showing the different flake regions and the corresponding stacking configurations on the device. **c**, PL maps measured from the monolayer on two void arrays. **d**, An AFM line-cut measured along a void array row comparing the topography of the three stacking cases. **e**, Finer PL map of the top void array shown in Figure S8c. Seven circular regions of equal areas were selected across different parts of the array for quantitative comparison. **f**, Total PL intensity extracted from each circular region. Colors of the data points indicate the corresponding circular region in Figure S8e. Scale bar: 5 µm.

Comment 4: Foundational electromagnetic mode analysis for Mie voids is also absent. Characterizing electric/magnetic multipole modes and the Kerker effect—the physical basis of directional emission in such systems—is essential to understanding the optical effects observed, especially for the unique complementary geometry of Mie voids. This work only presents phenomenological observations, with no in-depth mechanistic analysis to support its core conclusions.

Response to Comment 4: We thank the reviewer for this comment. In the revised manuscript, we address this point by including a detailed multipolar analysis of the emitted field (Section: Directional emission using Mie voids, Fig. 5g,h), which identifies the directional emission in terms of interference between electric and magnetic multipolar contributions of the void resonance. This provides a direct mechanistic explanation of the observed beaming. Further details are provided in our response to Reviewer 1, Comment 2.

Comment 5: Finally, experimental results lack sufficient statistical generalizability. Mechanically exfoliated WS_2 monolayers have inherent structural inhomogeneities (stress variations, defects, interfacial impurities) that significantly impact PL properties, yet the study only presents results from limited samples and measurement positions, with no systematic statistical analysis across multiple voids or samples. This limited sampling may introduce bias from random inhomogeneities, reducing the reliability and generalizability of the study's conclusions.

Response to Comment 5: We thank the reviewer for this comment. We agree that demonstrating reproducibility across multiple structures is important. The manuscript already includes measurements on multiple voids and independent samples.

In Fig. 4, we present PL measurements on multiple individual voids with varying diameters, showing consistent enhancement trends as a function of geometry. In addition, Fig. 5 reports measurements on a separate void-array sample, where a statistical analysis of the peak PL intensity (Fig. 5c) shows only minor variation between individual voids.

To further strengthen this point, we have added additional control experiments on independent void arrays in Supplementary Section 7, which show consistent PL enhancement behavior across different stacking configurations.

Taken together, these results demonstrate that the observed enhancement and directionality are reproducible across multiple voids and samples, and are governed by the void geometry rather than local inhomogeneities in the monolayer.

We thank the reviewers for their evaluation of our manuscript and for their insightful feedback. We have carefully considered the feedback and provided a point-by-point response to all comments. Our responses are shown in green, whereas all revisions made in the manuscript and SI are shown in red.

Reviewer #1

Comment 1: When I agreed to review the rebuttal version of this manuscript, I expected that the revised submission would contain substantial modifications addressing the concerns raised during the initial review process. In particular, given the significant criticisms expressed by Reviewers 2 and 3 regarding the impact and broader perspective of the work, I anticipated additional experimental evidence or a clearer demonstration of the manuscript's significance. However, while the text has been revised in several places, the main manuscript itself has changed only marginally, with most additions appearing in the Supporting Information.

I have carefully considered both the authors' responses and the revised manuscript. The responses are generally thoughtful and reasonable; however, my main concern is that these clarifications have not translated into corresponding improvements in the central message or impact of the manuscript itself. The extent of the rebuttal document, 19 pages in total, reflects the substantial nature of the questions raised during review. Nevertheless, the revisions do not, in my opinion, sufficiently address the key concerns regarding significance and positioning of the work.

Response to Comment 1: We thank the reviewer for carefully reassessing the revised manuscript. As requested during the first review round, we performed substantial additional simulations and experimental control measurements, which strengthen the physical interpretation and support the conclusions of the manuscript. These additions were important for substantiating the conclusions, but they did not change the central physical message of the work. We therefore incorporated the new multipolar decomposition into the main manuscript, as it provides additional physical insight into the experimentally observed directional emission, while the remaining additions were included in the Supporting Information because they primarily provide additional validation of the conclusions rather than alter the central narrative.

Comment 2: After reconsidering the manuscript in light of the first-round reports and the rebuttal, I arrive at a conclusion similar to that of Reviewers 2 and 3. The experimental results are interesting and technically sound, but I remain unconvinced that the work demonstrates the level of conceptual advance or broad impact expected for publication in Nature Communications. In particular, the manuscript does not clearly establish what distinguishes this approach from existing concepts or where it offers a decisive advantage over previously demonstrated systems. Since stronger enhancement factors have already been reported using solid-particle-based approaches, I believe the manuscript would benefit from a clearer experimental demonstration of the unique opportunities enabled by the present concept which is clearly missing.

I understand the authors' argument regarding the simultaneous enhancement of excitation and emission processes. However, given that the demonstrated performance appears less efficient than several existing approaches in the literature, I view this more as an important specialized contribution than as a broadly transformative advance.

Response to Comment 2: We thank the reviewer for sharing their perspective on the significance of the work. As discussed throughout the manuscript, the objective of this study is not solely to maximize photoluminescence enhancement, but to establish Mie voids as a nanophotonic platform for controlling light–matter interactions involving atomically thin emitters. In this framework, the key result is that the Mie void platform enables simultaneous optimization of excitation and emission processes while supporting directional emission. We believe the additional benchmarking against conventional dielectric resonators and the new mechanistic analysis included in the revised manuscript further clarify this central point.

Comment 3: Since the first review round, I have also become aware of two recently published papers that report closely related experiments and which, to the best of my knowledge, are not cited in the present manuscript: Light–matter interaction in van der Waals heterostructures with Mie voids (Advanced Photonics 8, 026002 (2026)) and Mie-Void-Enabled WS_2 Excitonic Emitters with Nanoscale Footprints (Nano Letters 26, 1888 (2026)). These publications further reduce the perceived novelty and impact of the present work. At the same time, I recognize that these studies may have been conducted on similar timelines, and therefore questions of priority and novelty are ultimately editorial considerations.

Comparing these recent publications with the current manuscript also influenced my assessment of the work's scope and contribution. The Advanced Photonics paper investigates both photoluminescence enhancement and second-harmonic generation while employing resonance tuning through systematic depth variation, thereby avoiding the rather unconventional normalization procedure used in the present manuscript. This comparison also highlights that the newly introduced fabrication method for depth control shown in the current manuscript, while technically interesting, appears somewhat peripheral to the manuscript's main focus on photoluminescence enhancement. Ultimately, the manuscript centers on two photoluminescence-related experiments, one addressing enhancement and one radiation control, which, although interesting, do not in my view reach the threshold of general significance required for publication in Nature Communications.

Response to Comment 3: We thank the reviewer for pointing out the two recent related publications. These works appeared during the review and revision process of our manuscript, and we have now cited them in the revised manuscript, together with other recent studies that have emerged in this rapidly developing area (Refs. 33–36 and 38).

While the primary scientific focus of the manuscript is on controlling light emission using Mie void resonators, the fabrication strategy constitutes an important enabling aspect of the work by providing independent control over the void diameter and depth. This capability is essential for accessing the geometrical parameter range in which excitation and emission processes are simultaneously optimized, thereby enabling the experimentally demonstrated enhancement and directional emission.

In the revised manuscript, we now cite the two papers mentioned by the reviewer as well as other recent studies on Mie voids that have emerged during the review and revision process of our manuscript (Refs. 33-36 and 38).

Comment 4: I am puzzled by the simulation results involving the Si disk. There is substantial literature precedent demonstrating enhanced emission in related systems, and it is therefore unclear why the presented simulations appear to indicate the opposite behavior.

Response to Comment 4: We thank the reviewer for this comment. Our simulations do not indicate that Si nanodisks are incapable of providing photoluminescence enhancement. On the contrary, they show substantial enhancement for appropriately chosen geometries. The purpose of the comparison is instead to demonstrate that, for an emitter located outside the resonator, the excitation and emission optima of the Si nanodisk generally occur at different geometries, making their simultaneous optimization considerably more geometry-sensitive than for the Mie void platform. To clarify this point, we have added the following sentences to Supplementary Section 4:

"Importantly, these simulations do not imply that Si nanodisks cannot provide PL enhancement. Rather, they show that high enhancement is achieved only within a relatively narrow region of the geometrical parameter space where the optima for excitation and emission overlap."

Comment 5: I do not fully understand the significance of the phasor representation introduced in Fig. 5h, which appears to be the only substantial addition to the main text. Its role and physical interpretation are only minimally explained, making it difficult to assess its relevance to the manuscript's central conclusions.

Response to Comment 5: We thank the reviewer for pointing out that the significance of the phasor representation was not sufficiently clear. The purpose of Fig. 5h is to complement the far-field radiation diagram shown in Fig. 5g by providing a visualization of how the individual multipolar contributions interfere along the forward direction. In particular, it makes explicit that the experimentally observed directional emission arises from constructive interference between several void-induced multipolar contributions and the background emitter field. To clarify this point, we have added the following clarifying sentence to the main text:

"The phasor diagram shows the relative amplitudes and phases of the individual multipolar contributions and illustrates how they interfere to produce the observed directional emission."

Reviewer #3

The authors have addressed the major concerns raised by the reviewers by providing additional calculation results. I believe that the manuscript is now suitable for publication in Nature Communications.

We sincerely thank the reviewer for their careful reassessment of our manuscript. We appreciate their positive evaluation of the revised work and their recommendation for publication in Nature Communications.
